# Sequence of Immunological Events During IgE‐Mediated Allergic Reactions to Food

**DOI:** 10.1111/all.70194

**Published:** 2025-12-24

**Authors:** N. A. Nagy, D. Schnoegl, V. Houghton, L. O'Mahony, A. F. Santos, E. F. Knol, A. Kuehn, T. Eiwegger

**Affiliations:** ^1^ Department of Infection and Immunity Luxembourg Institute of Health Esch‐sur‐Alzette Luxembourg; ^2^ Karl Landsteiner University of Health Sciences Krems Austria; ^3^ Peter Gorer Department of Immunobiology, School of Immunology and Microbial Sciences King's College London London UK; ^4^ Department of Medicine and Microbiology, APC Microbiome Ireland National University of Ireland Cork Ireland; ^5^ Department of Women and Children's Health (Pediatric Allergy), Faculty of Life Sciences and Medicine School of Life Course Sciences, King's College London London UK; ^6^ Children's Allergy Service, Evelina London Children's Hospital Guy's and St Thomas' Hospital London UK; ^7^ Center of Translational Immunology and Dermatology/Allergology University Medical Center Utrecht Utrecht the Netherlands; ^8^ Department of Immunology, Temerty Faculty of Medicine University of Toronto Toronto Ontario Canada; ^9^ Department of Pediatric and Adolescent Medicine University Hospital St. Pölten St. Pölten Austria; ^10^ Translational Medicine, Research Institute Hospital for Sick Children Toronto Ontario Canada

**Keywords:** basophils, food allergy, gastrointestinal tissue, IgE, immune mechanism, mast cells, peanut allergy, T2 responses

## Abstract

Food allergies (FA) represent a significant global health burden. Upon allergen re‐exposure, allergic patients exhibit a sequence of symptoms that vary in terms of affected organ systems, severity, time of onset and allergen reactivity thresholds. This review focuses on the sequence of immediate immunological and clinical events that occur during allergen exposure in individuals with FA. It highlights both clinical and immunological baseline risk factors and discusses recent advances in understanding the acute allergic response. We explore the implications of immune mechanistic knowledge for predicting clinical reactivity and therapy of FA. The current diagnostic gold standard, the oral food challenge (OFC), may help identify early biomarkers for patient stratification and provide insights into the immune dynamics underlying acute allergic reactions in vivo.

## 1 Introduction

Food allergies (FA) are an increasing global health concern, affecting up to 10% of children and adults in Europe, Australia and North America [1, 2, 3, 4, 5, 6, 7, 8]. Since FA is the leading cause of anaphylaxis in children and adolescents, the number of anaphylactic reactions is consequently increasing [9, 10, 11, 12]. Cow's milk, peanuts, tree nuts and shellfish are the major culprit foods of near‐fatal reactions [10, 13, 14, 15, 16, 17]. FA significantly impacts the quality of life for patients and their caregivers, affecting mental health, reducing social interactions, increasing the cost of living and negatively impacting overall well‐being. Despite a relatively good understanding of how allergen‐specific IgE contributes to food reactions, the sequence of immune events that drives a systemic allergic reaction to a food allergen is poorly understood. However, a deeper mechanistic understanding of these interactions is required to identify high‐risk populations more prone to experience life‐threatening reactions and to discover novel approaches to prevent, diagnose and treat food allergies [18, 19, 20].

This review aims to illustrate the sequence of events that happen during an IgE‐mediated food‐allergic reaction at the molecular level and list co‐factors that impact the severity of an allergic reaction based on available evidence.

## 2 Baseline Risk Factors Associated With Severe Allergic Reactions

**FIGURE 1:**
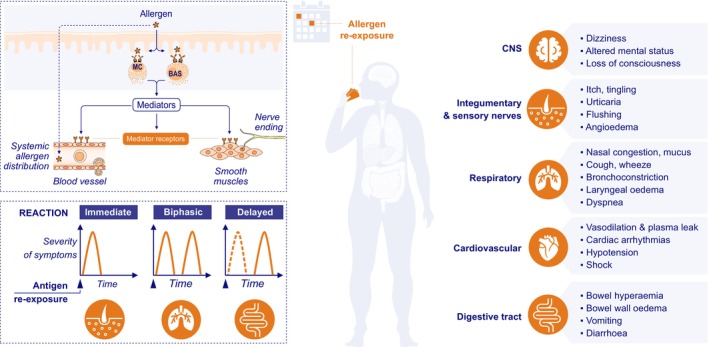
Organ systems affected by allergen re‐exposure in the acute food‐allergic response. Upon food allergen re‐exposure, a rapid local response may occur through nerve endings in the skin and oral mucosa, interacting with mast cells, basophils and their mediators. Food allergens are swiftly absorbed into the bloodstream, triggering systemic degranulation of mast cells and basophils. Affected organ systems include the central nervous system, the skin, respiratory and cardiovascular systems, and the digestive system. Symptoms may appear quickly, in a biphasic pattern, or be delayed, with integumentary symptoms manifesting early and gastrointestinal symptoms often emerging later. Orange stars, food allergens; BAS, basophil; CNS, central nervous system; MC, mast cell.

Although many FA patients experience typical symptoms, allergic reactions can vary in timing and severity (Figure 1), even in cases of exposure to a comparable amount of allergen from the same source [21, 22]. This heterogeneity has been linked with co‐factors that modify the severity of a reaction such as exercise, medications (ACE‐inhibitors, beta‐blockers, NSAIDs), asthma, infections, alcohol consumption, sleep deprivation, starvation, hot shower or bath, stress and many more. The relative impact of each co‐factor varies at an individual level and may change over time. So far, no single biomarker has been identified to effectively predict the risk of reaction severity [22, 23, 24].

Some risk factors are known to increase the likelihood of severe systemic reactions (Box 1). Underlying mast cell (MC) disorders, such as mastocytosis [25] and hereditary alpha‐tryptasemia (HαT) [26] are linked to food‐driven anaphylaxis and FA in general. In HαT, food‐triggered anaphylaxis is significantly more common. A case study showed a lower threshold during oral food challenge (OFC) in allergic siblings with HαT compared to an allergic sibling with the conventional tryptase genotype [27]. Unlike in hymenoptera allergy, MC‐associated pathologies are not directly associated with FA reaction severity [23, 28]. Sensitisation to some allergens makes systemic reactions more likely; however, the extent of sensitisation (abundance of sIgE) does not predict the threshold or the severity of the reaction.

BOX 1 *Baseline risk factors*

– Adolescent or young adult age
– High allergen‐specific IgE to selected allergens
– Mastocytosis
– Hereditary alpha‐tryptasemia (HαT)
– Severe asthma

*Augmentation factors*

– Exercise
– Medications (e.g., NSAIDS, Beta‐blockers)
– Alcohol
– Sleep deprivation
– Stress
– Empty stomach
– Infections
– Amount of allergen
– Uncontrolled asthma
– Menstrual cycle

### 2.1 Humoral and Soluble Markers as Risk Factors

While allergen‐specific IgE levels are used as an indicator of FA, they do not predict severity. Sensitisation to some allergen families, such as seed storage proteins; in particular 2S‐ albumins or LTPs, is linked to systemic reactions [29, 30, 31, 32, 33, 34]. Reactivity to an allergenic source is not only influenced by sIgE levels but also by affinity and avidity [35, 36], epitope diversity and epitope proximity (Box 2) [37]. High IgE affinity and avidity differed between peanut‐allergic versus peanut‐sensitised tolerant individuals [35, 36]. In contrast, high levels of IgG binding can compete with IgE and therefore reduce effector cell degranulation [38, 39]. IgE binding and signalling strength are affected by post‐translational modifications, such as glycosylation of its constant region [40, 41]. Similarly, IgG‐mediated anti‐IgE responses depend on glycosylation of IgE‐immune complexes and further regulation of IgE levels [42].

Mediators released by effector cells such as tryptase and platelet‐activating factor (PAF) have been explored as severity biomarkers. PAF can bind to PAF receptors on endothelial and various immune cells, including monocytes, macrophages and neutrophils. It can induce bronchial smooth muscle contraction and have angiogenic properties. PAF may induce vascular hyperpermeability and injury, thrombotic responses, hypotension, and activate endothelial production of nitric oxide [43]. These vascular changes can contribute to airway oedema, hypovolemia and circulatory collapse, during which a falling heart rate despite hypotension represents a dangerous complication and can lead to an anaphylactic shock with potentially fatal consequences [44, 45].

BOX 2 *Baseline*:

– BAT by CD63 and/or CD203 expression to baked products [46, 47].
– BAT % CD63 pos basophils peanut/anti‐IgE [48, 49, 50].
– BAT % CD63 pos basophils after FcERI od fMLP stimulation [51].
– Basophil expression of the FcεRI co‐receptor CD300c [52].
– Platelet‐activating factor acetylhydrolase activity (PAF‐AH) [53, 54].
– IgE affinity and avidity [35, 36].
– IgE modifications (e.g., glycosylation) [55, 56].
– Composite models [51].
– Salivary IgE and IgG4/IgE ratio [50, 57].
– α‐tryptase isoform copy number [58].

*Acute biomarker during reaction*:

– Tryptase (1.2 times baseline plus 2 ng/mL) [59, 60].
– Histamine [61].
– Platelet‐activating factor [54, 60, 62].
– Urinary PGD_2_ [60, 63, 64].
– IL‐6, 10 and soluble TNFRI [65, 66, 67].

Additionally, PAF is an important regulator in different inflammation‐associated processes such as the expression of adhesion molecules and leukocyte recruitment [68, 69]. Its concentration is regulated by PAF acetylhydrolase (PAF‐AH). Several studies in peanut, cashew nut or insect venom allergies suggest that a lower degree of PAF inactivation by PAF‐AH may enhance the severity of allergic reactions [53, 54, 68, 70, 71], and targeting the PAF pathway has been suggested as a potential novel therapy option in both allergic and non‐allergic inflammatory conditions [72].

### 2.2 Cellular Markers as Risk Factors

Mucosal inflammation may predispose higher clinical reactivity, and allergic individuals display differences in their cellular immune compartment, which resolve when outgrowing FA [73, 74, 75, 76]. These may potentially serve as biomarkers for allergic reactions or predictors of severity.

Mucosal MCs are important effectors during allergic reactions; their numbers rapidly increase during mucosal allergic inflammation and correlate with the severity of symptoms [77]. Different MC phenotypes have recently gained attention, as it was shown that their phenotype in anaphylactic patients differs from the MC phenotype in sensitised patients. MCs from anaphylactic patients show high degranulation and production of PGD2, IL‐8 and GM‐CSF, while MCs from sensitised patients are characterised by production of TGF‐β and CCL2 [78]. Additionally, different MC subtypes have been associated with different manifestations of food allergies in mice: while connective tissue mast cells, as defined by their expression of MMCP‐7, were activated during systemic anaphylaxis, a mouse model of gastrointestinal manifestation of FA was associated with an increased activation by MMCP‐1 producing MCs in their intestine [79]. However, their role is not yet completely understood, as MC activation during OIT can lead to allergic reactions and impair the establishment of tolerance, but some studies suggested that they can also be beneficial for desensitisation during the early phase of OIT [80].

Allergen‐responsive effector T‐cells can be identified by means of tetramer staining or the upregulation of the activation marker CD154 upon allergen exposure. Allergen‐specific Th2A cells, a terminally differentiated pathogenic Th cell subset, have been linked to FA. These cells are characterised by the expression of CRTH2 and ST2 and the ability to produce and release several type 2 (T2) cytokines at the same time [81, 82]. The frequency of Th2A cells and their activation increase after allergen exposure in peanut allergic patients during a food challenge [81, 83, 84, 85, 86, 87] and their numbers decrease during successful OIT [88]. While regulatory T‐cells (Tregs) may be altered in FA patients [89], their importance in FA remains controversial. In vitro evidence suggests that certain regulatory subsets can directly suppress MC degranulation, e.g., through cognate OX40‐OX40L interaction [90]. However, other subsets may potentially support a Th2 bias, which is reversed by OIT [91, 92, 93, 94]. For example, peanut‐specific Treg showed a Th2‐like phenotype and IL‐4 production, which was inhibited by OIT [93]. Additionally, reduced baseline abundance of both naïve and terminally differentiated CD8+ T‐cells was associated with sustained unresponsiveness to peanut OIT. These CD8+ T cells can interact with Th2 cells, enhancing allergic inflammation in desensitised patients [95, 96].

Alarmin‐triggered ILC2s can modify the activation threshold of effector cell populations and interact with T follicular helper type 13 cells (Tfh13) [97]. Specifically, Tfh13 have been implicated in the generation of high‐affinity IgE production by plasma cells that are required for severe FA [97, 98, 99]. Studying human IgE‐plasma cells in FA is highly biopsy‐dependent, as up to 80% of them are estimated to reside in the GI tract, especially in GALT of the small intestine [100, 101, 102]. Indeed, a study sampling 19 peanut‐allergic patients found IgE+ B cell clones with overlapping Ara h 2‐specific clonotypes specifically enriched in the duodenum [103]. These B‐cells had a plasma cell phenotype. Additionally, two very recent studies identified CD23^hi^, IL‐4Rα^hi^ and CD32^low^ IgG‐producing B‐cells as precursors for IgE+ plasma cells in birch, house dust mite and peanut‐allergic patients. This points to a common underlying pathogenic mechanism and sheds light on discrepancies between circulatory and tissue presence [104, 105]. However, further biopsy studies are needed to identify immunological predispositions for severe reactions.

### 2.3 Microbial Metabolism as Co‐Factor

The individual risk for FA can be impacted by nutrition, microbiome composition and its function, which in turn affects the immune system and the likelihood to generate a symptom‐evoking response. Supplementation of omega 3 fatty acids and a high fibre diet have beneficial effects on immune cell differentiation, production of anti‐inflammatory cytokines, IgE levels and gut microbiome diversity [106, 107, 108, 109, 110]. Salivary bacterial α‐diversity, *Rothia aeria* and fecal bacteroides correlated with low threshold responses during OFC [111, 112, 113]. Short chain fatty acids (SCFAs) in FA have been reviewed recently [114]. SCFAs are produced by gut microbiota protect against allergic diseases, including atopic dermatitis, asthma and FA, potentially by enhancing the expression of genes associated with gut barrier integrity and tolerogenic cytokines; however, the exact mechanisms are not fully understood [114, 115, 116].

## 3 Sequence of Immune Events During the FA Reaction

### 3.1 Food Allergen Ingestion and Uptake

**FIGURE 2:**
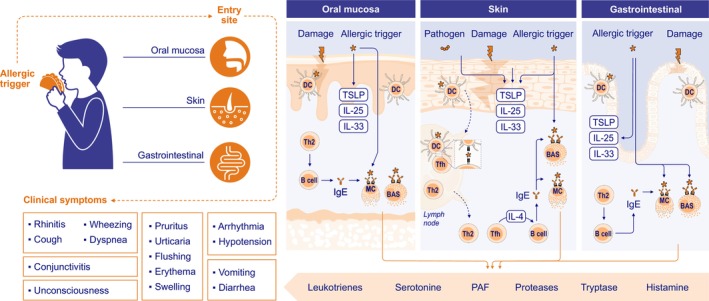
Allergen uptake and entry via epithelial barriers. The oral route is the primary site where allergens initiate an acute immune response. However, allergens may also trigger skin reactions upon direct contact, respiratory symptoms via inhalation, as well as gastrointestinal symptoms. Circulatory distribution may activate effector cells at distant sites. A compromised epithelial barrier can exacerbate symptoms (damage), involving heightened presence of alarmins, T2 cytokines and stronger degranulation of effector cells. Orange stars, food allergens; BAS, basophil; DC, dendritic cell; MC, mast cell; PAF, platelet‐activating factor; Tfh, T follicular helper cell; TSLP, thymic stromal lymphopoietin.

The gastrointestinal (GI) tract, skin and respiratory system can all serve as primary entry points for allergens (Figure 2). Sensitisation to food allergens is thought to occur through the skin, the respiratory tract or the GI tract due to a loss of oral tolerance during the critical weaning period in early life, in a complex interplay between epithelial barrier, mucosal immunity, food components and gut microbiota [117, 118, 119, 120].

Re‐exposure to food allergens often occurs in the oral cavity, where severe anaphylactic symptoms can start within seconds. In these cases, a reaction may begin before allergens even reach the GI tract, underscoring the importance of studying immediate immune interactions in the oral mucosa. The oral epithelial barrier consists of several regions with varying permeability: the keratinised stratified squamous epithelium of the hard palate and gums, the non‐keratinised stratified epithelium underneath the tongue and inner cheeks and the dorsal epithelial surface of the tongue [121]. Unlike the intestinal epithelium, the oral epithelial barrier is highly selective, preventing uncontrolled antigen penetration. Nevertheless, drugs are readily absorbed, especially at richly vascularised non‐keratinised surfaces [122]. Interestingly, some FA patients exhibit delayed or biphasic symptoms upon re‐exposure—it is possible that variations in oral mucosal absorption can lead to different patient endotypes. One of the earliest studies highlighting the unique characteristics of peanut allergens demonstrated that, within just 10 min of peanut exposure—without even swallowing—non‐allergic individuals had detectable levels of peanut allergens in their blood sufficient to trigger degranulation of passively sensitised basophils [123]. In a follow‐up study, Mose et al. further examined allergen absorption kinetics in non‐allergic adults using an in vivo passive cutaneous anaphylaxis test. In this test, non‐allergic subjects were intradermally sensitised with peanut‐allergic sera, after which they ingested peanut, and the time to onset of erythema at the skin site was measured. Ingestion of peanut protein led to detection in the serum within 5 min, peaking at 1–4 h and circulating until 48 h post ingestion [124]. Median erythema reaction time increased when using more diluted IgE sera during sensitisation and decreased with higher oral peanut doses upon challenge [125]. Moreover, wheal size also increased depending on IgE serum concentrations and the ingested allergen dose. In a similar manner, Bernard et al. developed a blood pre‐treatment protocol enabling detection of the major peanut allergen Ara h 6 in the bloodstream after low‐dose ingestion and showed that allergen bioaccessibility varies with food matrix and individual allergic status [126]. Notably, peanut‐allergic individuals exhibited higher and earlier Ara h 6 absorption compared to non‐allergic controls, highlighting potential implications for reaction severity and threshold variability.

Characteristics of the allergen also determine absorption kinetics along the GI tract [122]. A study by Williams et al. assessed the penetration of glycerinated allergen extracts and commercially available food powders in a human epithelial cell line resembling the oral barrier, finding that glycerinated egg extract penetrated the barrier more effectively compared to commercially available egg food powder. Of note, penetration rates increased with incubation time for all tested extracts (peanut, cashew, egg, cow's milk and sesame) [127]. Bio‐accessibility of allergens may also differ in saliva. For instance, the major peanut allergens Ara h 2 and Ara h 6 are released more rapidly from roasted peanut flour compared to Ara h 1 and Ara h 3 in buffers mimicking human saliva [128, 129, 130]. Moreover, salivary components can also serve as biomarkers for FA diagnosis and patient stratification. In a phase II peanut OIT study, peanut‐specific IgG4 and IgA were detected in saliva [131]. Additionally, salivary IgE levels before the placebo‐controlled food challenge correlated with reaction thresholds in peanut‐allergic patients [57]. Children with high peanut‐specific salivary IgE had a higher odds ratio to be low threshold responders at the first challenge, while children with a high peanut IgA‐to‐IgE ratio or peanut IgG4‐to‐IgE ratio had higher threshold responses. Furthermore, children with high pre‐challenge peanut IgG4 in saliva also had a lower chance of severe reactions during the challenge. Notably, there was only a modest correlation with serum sIgE levels, indicating that saliva measurements may better predict threshold dose and reaction severity.

Differential digestive timing may influence immediate versus delayed reactions, as demonstrated by a study comparing basophil histamine release assay (BHRA) after pork‐kidney smoothie (alpha‐gal) ingestion versus peanut [132]. BHRA max release happened within 30 min for peanut versus 3–4 h after pork kidney consumption, suggesting that delayed FA reactions as seen in alpha‐gal syndrome may be dependent on absorption or allergen release kinetics.

Despite these observations, the link between rapid oral absorption and an increased risk of severe allergic reactions remains debated. In mice sensitised to Japanese cedar pollen extract, rapid allergen uptake via cell‐penetrating albumin reduced symptoms of nasal scratching [133]. In contrast, in already sensitised individuals, rapid oral allergen penetration may lead to breakdown of the oral barrier and predispose to anaphylaxis. Studies investigating repeated exposure to the legume protein profilin revealed that patients developed progressive remodelling of the oral mucosa. This was marked by a decrease in cell junction protein expression, resulting in a more permeable epithelial barrier, and an increase in angiogenesis within the exposed tissue [134, 135, 136, 137]. These changes were particularly pronounced in patients with a history of severe reactions, correlating with an upregulation of IL‐33 and the presence of IL‐33–dependent ILC2s [138, 139, 140]. Decreased expression levels of the tight junction protein Claudin‐1 have been documented in the oral mucosa [141] of profilin‐allergic patients, in the oesophagus of patients with eosinophilic esophagitis [142] and in the intestines of a rat model of FA [143]. Moreover, mutations in skin barrier proteins such as serine protease inhibitor Kazal type 5 and filaggrin have been linked to the progression from atopic dermatitis to FA [144]. However, despite these associations, markers of epithelial barrier integrity—including those mentioned above—have not been shown to reliably predict clinical reactivity in FA [145].

The oral cavity houses lymphoid structures, including the Waldeyer's ring and tonsils [146]. Immune reactions in the oral cavity may happen independently, compartmentalised from the rest of the GI tract [147]. Thus, they may play a role in the sequence of events during FA reactions [148, 149]. Transcriptomic profiles of tonsillar samples were unique to food‐allergen‐sensitised patients [150]. As mentioned above, a subset of FA patients present with delayed or biphasic reactions to food allergens. Whether early reactions can be explained by immune interactions within the oral mucosa, including mechanisms of enhanced cross‐epithelial transport of food allergens [151], remains unresolved.

### 3.2 Early‐to‐Late Immune Events Upon Food Allergen Exposure

**FIGURE 3:**
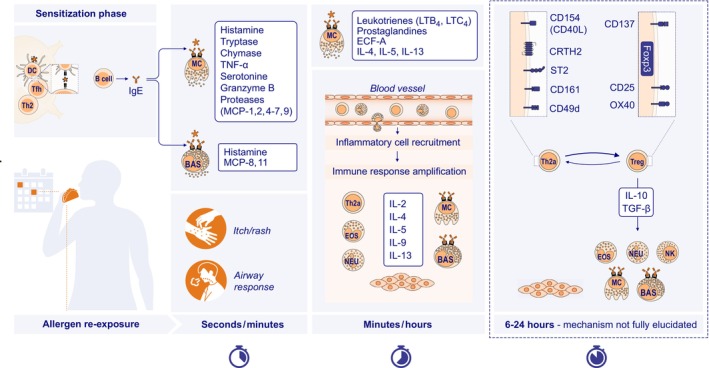
Immune response sequence following allergen re‐exposure. Re‐exposure to food allergens leads to FcεRI cross‐linking and downstream signalling in mast cells or basophils, leading to rapid degranulation and immediate symptoms (from left to right, first and second panel). Histamine and other mediators are released within minutes, while leukotrienes appear within hours (second and third panel). Cytokine release further amplifies the immune response, attracting more effector and innate immune cells (third panel). Responses beyond 6 h remain unclear (fourth panel, indicated by dashed margin) but may involve (allergen‐specific) Th2a cells expressing CD154, CRTH2, ST2, CD161, CD49d and concomitant production of more T2 cytokines (fourth panel), together with Tregs entering the cascade later through a potential feedback loop between Th2a and Treg cells (fourth panel). Orange stars, food allergens: BAS, basophil; ECF‐A, eosinophil chemotactic factor of anaphylaxis; EOS, eosinophil; LTB_4_, leukotriene B4; LTC_4_, leukotriene C4; MC, mast cell; MCP, mast cell proteases; NEU, neutrophil.

Immediate reactions in FA upon re‐exposure are orchestrated by basophils and MCs, whose mediators rapidly accelerate the inflammatory cascade. Capturing the sequence of events during acute FA reaction, together with the cells and compounds that signify them, is important in the search for novel FA biomarkers (Figure 3). In vivo studies carried out in animal models, together with key studies monitoring immune changes during OFC in FA individuals, have delivered important insights in the past years.

#### 3.2.1 Seconds to Minutes Post‐Allergen Ingestion

**FIGURE 4:**
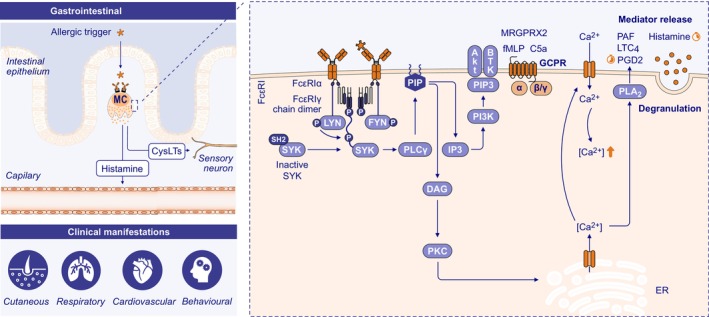
Direct signalling events in basophils and mast cells leading to inflammatory mediator release. Allergen binding to IgE on FcεRI receptors triggers receptor cross‐linking, ultimately resulting in mediator release affecting several organ systems including immediate, proximal neuronal symptoms as well as distal effects (left panel). Intracellular events after cross‐linking are depicted in the right panel. Cross‐linking brings the immunoreceptor tyrosine‐based activation motifs (ITAMs) of the β and γ chains into proximity. This activates Src kinases, which recruit spleen tyrosine kinase (Syk) through its SH2 domains. Both phospholipase gamma (PLCγ) and phosphoinositide 3‐kinase (PI3K) pathways are activated. PLCγ cleaves phosphatidyl‐4,5‐biphosphate (PIP_2_) into inositol triphosphate (IP3) and diacylglycerol (DAG), with IP3 stimulating calcium release from the endoplasmatic reticulum (ER) and DAG activating protein kinase C (PKC). PI3K generates PIP3, recruiting Akt and Bruton's tyrosine kinase (BTK) to the plasma membrane. Intracellular calcium release activates calcium release‐activated calcium (CRAC) channels, allowing sustained calcium influx critical for microtubule reorganisation and granule fusion, leading to degranulation after approximately 3 min. Basophils and mast cells also release lipid mediators such as leukotriene C4 (LTC_4_), platelet‐activating factor (PAF) and prostaglandin D2 (PGD_2_). Additionally, G‐protein‐coupled receptors (e.g., C5a, fMLP, MRGPRX2) rapidly activate basophils and mast cells, inducing faster degranulation (10–20 s) than FcεRI cross‐linking. Orange star, allergen; Ca^2+^, calcium; CysLTs, cystenyl leukotriene receptors; fMLP, N‐formyl‐methionyl‐leucyl‐phenylalanine; MRGPRX2, Mas‐related G‐protein‐coupled receptor X2; PLA_2_, phospholipase A2; clocks indicate mediator release timing.

Cellular activation occurs as a consequence of intracellular signalling events. When a multivalent allergen binds to IgE antibodies pre‐bound to high‐affinity FcεRI receptors on the surface of basophils or MCs, it induces receptor cross‐linking and clustering (Figure 4) [152, 153]. Per cell, there needs to be at least 100 of these cross‐linking events to initiate intracellular signalling [154, 155]. The structural receptor rearrangements bring the immunoreceptor tyrosine‐based activation motifs (ITAMs) of FcεRI's β and γ chains into proximity, enabling phosphorylation by membrane‐associated Src family kinases (primarily Lyn and Fyn) [156, 157, 158]. The phosphorylated ITAMs then serve as docking sites for spleen tyrosine kinase (Syk), which is recruited through its SH2 domains. Syk activation subsequently initiates two signalling pathways, the phospholipase gamma (PLCγ) and phosphoinositide 3‐kinase (PI3K) pathways [159, 160]. PLCγ cleaves plasma membrane phosphatidyl‐4,5‐biphosphate (PIP_2_) into inositol triphosphate (IP3) and diacylglycerol (DAG). IP3 triggers calcium release from endoplasmic reticulum (ER) stores through IP3 receptors, while DAG activates protein kinase C (PKC). The PI3K generates PIP3 which recruits Akt and Bruton's tyrosine kinase (BTK) to the plasma membrane [161]. The intracellular calcium release stimulates calcium release‐activated calcium (CRAC) channels, resulting in sustained extracellular calcium influx, which is crucial for microtubule reorganisation and granule fusion [162]. That is combined with the PKC pathway finally triggering the fusion of the granules with the plasma membrane resulting in degranulation, which occurs after about 3 min.

Next to degranulation also potent lipid mediators are released by basophils and MCs. Basophils release leukotriene C4 (LTC_4_) and platelet activation factor (PAF), while MCs release prostaglandin D2 (PGD_2_) and PAF [163, 164, 165, 166]. Signalling events for the release of these mediators share most of the players from the degranulation‐signalling cascade described above, but only specifically use activation of phospholipase A2 (PLA_2_), which is induced by increase in cytosolic calcium and PKC [167, 168]. PLA_2_ induces the release of arachidonic acids from membrane phospholipids leading to stepwise formation of LTC_4_, PAF or PGD_2_. There are some subtle differences between signalling for degranulation versus the lipid mediator release. For basophils it has been described that G‐protein receptor activation by C5a results in rapid degranulation, but for LTC_4_ release it needs an additional pre‐exposure to interleukin 3 [164]. Parallel to these signalling events leading to the degranulation and mediator release, there is also Mitogen‐Activated Protein Kinase (MAPK) and Nuclear Factor kB (NF‐κB) activation. These activate extracellular signal‐regulated kinase (ERK), p38 and c‐Jun NH2‐terminal kinases (JNK) pathways that are predominantly involved in upregulating cytokine synthesis, which are secreted much later, at least 6–24 h after activation [169, 170, 171]. Another rapid route for activating basophils or MCs is through ligands of G‐protein‐coupled receptors (GPCRs), such as the anaphylatoxin C5a or peptides like fMLP. In MCs, activation can also occur via the Mas‐related GPCR member X2 (MRGPRX2) [172]. Ligands for MRGPRX2 include certain drugs and neurotransmitters, such as substance P. Stimulation of these GPCRs induces degranulation much faster than FcεRI‐cross‐linking, typically within 10–20 s. However, no food‐derived component has yet been identified that directly activates these receptors. Still, indirect effects‐ such as neuropeptides released by gut neurons during an FA reaction‐ may activate MRGPRX2 and thereby contribute to rapid MC degranulation [173].

Interestingly, even though eosinophils and basophils appear to be increased at baseline, their abundance in blood decreases during an anaphylactic reaction [70]. Next to, or sometimes even independent of degranulation, basophils also migrate to affected tissues. This has implications when assessing peripheral blood basophils following allergen challenge because the most responsive basophils might leave the bloodstream and become part of the margination pool [174, 175, 176].

#### 3.2.2 Minutes to Hours Post‐Allergen Ingestion

Human in vivo studies assessing allergic effector cell activation are scarce. Activation of immune cells from whole blood of six peanut‐allergic patients and three healthy controls was assessed 15 or 30 min after peanut extract stimulation, using mass cytometry [177]. Basophils were the most responsive cells in allergic patients, responding minimally in healthy controls. Basophils from FA patients uniquely upregulated CD61 and other receptors that fostered interaction with platelets and had increased phosphorylation of p38 and pERK. Physical interaction with platelets was confirmed using imaging cytometry, suggesting an FA‐reaction‐relevant mechanism that requires further exploration in future studies.

The role of innate cells, other than MCs and basophils, in reinforcing the acute allergic cascade is increasingly recognised in transcriptomic and multiparameter cytometry studies. Transcriptome analysis of genes differentially expressed during peanut OFC revealed an increased signature of neutrophils and macrophages 2 h after challenge, accompanied by a decrease in gene expression related to naïve CD4+ T‐cells 4 h after challenge [178]. A follow‐up transcriptomic and epigenomic study identified 318 differentially expressed genes and 203 CpG sites, which correlated with reaction severity (peanut severity genes and CpG sites). Yet again, deconvolution analyses identified neutrophil signatures positively associated with reaction severity [179]. An increase in whole blood neutrophils, accompanied by a reduction in eosinophils, basophils and lymphocytes at reaction onset compared to baseline was similarly observed in 81 OFC+ children suffering from food protein‐induced enterocolitis syndrome [180].

#### 3.2.3 Several Hours Post‐Ingestion and Beyond

Studies combining OFC with in vitro PBMC stimulation indicate that allergen‐specific memory T‐cells enter the FA reaction cascade at later time points (Figure 3). While their role in initiating the acute response is most likely negligible, these T‐cells may serve as markers of clinical reactivity and play an important role in contributing chronic allergic inflammation at the sites of exposure. Indirect evidence is provided by clinical trials suggesting significantly less GI‐related symptoms upon OIT to peanut under anti‐IL4Rα blockade in the absence of significant reactivity threshold modification in endpoint OFC [181, 182]. Klueber et al. reported that during peanut OFC, circulating frequencies of Th2 cells, memory Tregs and activated NK cells were lower in OFC+ children compared to OFC‐ children, suggesting acute migration of these subsets into tissues [183].

T‐cells provide an interesting in vitro model to study allergies, as allergen‐specific T‐cell and TfH polarisation reflects the allergic status [85, 184]. Allergen‐specific T‐cells can be identified by allergen stimulation with CD154 upregulation (in general, 6 and 18 h timepoints are used) or the more complex method of MHC II tetramer staining to identify epitope‐specific T‐cells [91, 185, 186]. Both methods have proven to be appropriate. These specific T‐cells can be further characterised via these methods based on cytokine production and surface marker profiles, providing insights into the nature of allergen‐specific responses [84].

In peanut‐allergic children, CD154+ T effector cells, considered equivalent to activated allergen‐specific Th2A cells [81, 82, 185], were elevated, producing more IL‐13 and correlating with sIgE and BAT measurements [187]. These allergen‐specific T‐cells also exhibited increased expression of skin‐homing markers, suggesting their role in targeting affected tissues. Further research in the CoFAR cohort from peanut‐allergic children revealed that allergen‐specific T‐cells were detectable as early as 6 h post in vitro PBMC stimulation, while Tregs only upregulated CD154 at 18 h, suggesting their delayed response in the allergic cascade [84, 85]. CD154+ allergen‐specific effector cells had patient‐stratifying potential, as they were elevated in patients with peanut allergy reacting to a single dose of 300 mg or less, but not in high‐threshold individuals. Moreover, higher clinical reactivity to peanut during OFC correlated with increased allergen‐specific T‐cells showing T2 functionality, supporting the idea that effector T‐cells do appear during re‐exposure, followed by a late wave of Tregs [84, 85, 188, 189]. The CoFAR authors propose a model in which peanut‐specific effector T‐cells are activated first, leading to a subsequent wave of non‐allergen‐specific, IL‐2‐mediated activation of Tregs (Figure 3). In their follow‐up study on peanut‐allergic infants, the authors confirm that allergen‐specific T‐cell responses at 6, 24 and 48 h post‐stimulation align with OFC data, showing increased T2 cytokines and a rise in Th2A cells and OX40+ Tregs 24 h and 10 days post‐challenge, respectively [185]. Treg responses, in turn, may be useful for stratifying naturally tolerant and allergic individuals [190, 191]. Earlier studies corroborate the Th2 profile of proliferating allergen‐specific T‐cells, which can be detected even upon stimulation with highly digested or mutated peanut allergens [192, 193, 194]. The down‐modulation of Th2A cells during OIT further supports their potential as biomarkers [86, 92, 195]. However, this potential may differ across FA subtypes. While data on peanut allergy is quite consistent, this is controversial for egg allergy, possibly reflecting differences in persistence versus natural outgrowth [83, 185, 196, 197]. Unfortunately, despite these advancements, the complexity of T‐cells still limits their clinical utility for decision‐making.

### 3.3 Neuroimmune Interactions During the FA Reaction

An additional feature of FA reactions that has recently gained attention is the bidirectional communication between the immune system, particularly allergy effector cells, and the peripheral and central nervous systems. The close proximity of MCs [198, 199] and eosinophils with neurons [200, 201] has been described across the literature in the context of allergy, indicating the potential for local signalling to neurons. In FA models specifically, intestinal anaphylaxis in hen's egg albumin‐sensitised rats led to increased firing of mesenteric afferent neurons within 30 s of allergen exposure [202]. The study alludes to the MC source of this effect, with its abolishment following blockade of the histamine H1 and serotonin 5‐HT3 receptors. As well as histamine and serotonin, the MC mediators tryptase [203] and chymase [204] have been shown to facilitate MC‐neuronal communication in allergy. Importantly, these neuro‐immune interactions in the context of FA appear to have direct implications for symptomologies associated with FA reactions. Specifically, tryptase was shown to lead to oedema in the raw paw via release of calcitonin gene‐related peptide (CGRP) [203], whereas chymase was involved in the thermoregulatory neural circuit response via TRPV1+ neuronal signalling [204]. Furthermore, leukotriene synthesis has been highlighted as being involved in the emergence of allergen aversion behaviours in ovalbumin‐sensitised mice [205, 206].

Interestingly, neurons have also been shown to be capable of communicating with MCs. In ovalbumin‐sensitised mice, vagus nerve stimulation was shown to ameliorate duodenal tissue inflammation; an effect that was accompanied by a reduction in the number of MCs and their activation [207]. Again, this communication appears to be involved in the experience of allergic reaction symptoms, with vagotomy being shown to prevent anaphylaxis in passive‐IgE‐sensitised mice [208]. However, another study revealed that vagal nerve stimulation led to amelioration of allergic diarrhoea in ovalbumin‐sensitised mice [209], demonstrating the need for further research to elucidate the specific role of neuroimmune interactions in the experience of FA symptoms.

Beyond neuronal‐immune cell interactions in the context of FA, neurons may also directly sense food antigen. The expression of functional FcεRI has been demonstrated in superior cervical ganglion (i.e., sensory) [210] and myenteric [199] neurons, with the latter demonstrating elevated [Ca2+]i after IgE‐antigen stimulation in an in vitro system [199]. Papain, the cysteine protease enzyme present in papaya, was shown to directly activate TRPV1+ neurons, leading to itch and pain behaviours in mice [211]. Moreover, papain‐induced neuronal release of Substance P triggered dendritic cell (DC) migration to the lymph node, where they initiated Th2‐cell differentiation [211], demonstrating a role of sensory neurons in allergen sensing and the subsequent immunological response. Another study revealed that 20% of dorsal root ganglion neurons responded to papain, and that this response was primed by a γδ T‐cell–IL‐3 signalling axis [212]. However, to date, the direct sensing of allergen by neurons has only been demonstrated in proteolytic allergens, with these two studies demonstrating the protease‐dependent nature of these effects [211, 212]. Whether neurons are capable of sensing allergens that lack protease activity remains to be determined.

## 4 Models to Study Food Allergies

### 4.1 Human Ex Vivo and In Vitro Models

**FIGURE 5:**
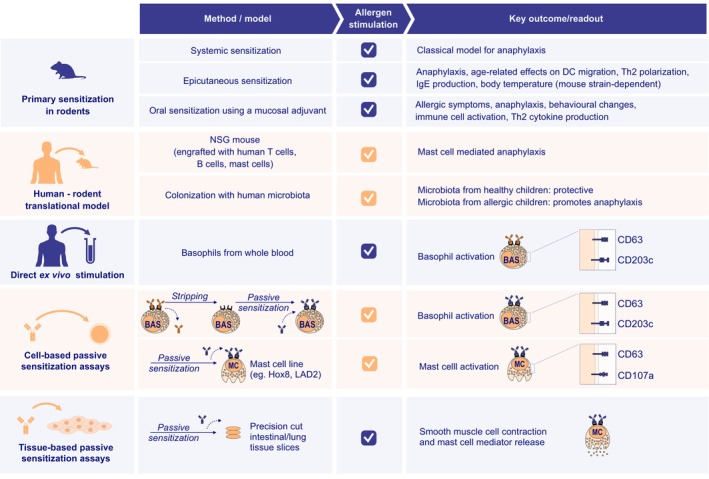
Models to study food allergies. To study IgE‐mediated anaphylaxis and immune cell activation in rodents, sensitisation to food allergens is typically induced intraperitoneally, epicutaneously, or orally, in combination with a mucosal adjuvant. Alternatively, human‐rodent translational models—which are significantly more complex—have been reported using severe combined immunodeficient mouse models or mice expressing a humanised FcεRI. Both passive and active sensitisation approaches are reported. Cell‐based in vitro or ex vivo models use either basophils from allergic individuals or basophils and mast cell lines passively sensitised with patient sera. Expression of activation/degranulation markers such as CD63 and CD203c is measured via flow cytometry following allergen‐specific stimulation. Additionally, passively sensitised precision‐cut intestinal or lung slices show smooth muscle cell contractions and mast cell mediator release after allergen stimulation. BAS, basophil; DC, dendritic cell; MC, mast cell.

To explore the sequence of allergic reactions in vitro and ex vivo, effector cell activation can be studied using in vitro assays such as basophil or MC activation tests (BAT or MAT, respectively) (Figure 5). BAT uses whole blood and assesses responsiveness to allergen extracts or individual allergens based on the upregulation of activation markers such as CD63 or CD203c. The outcome is measured by flow cytometry. BAT seems more sensitive and specific than patient history, SPT and IgE measurements and effectively distinguishes allergic from non‐allergic non‐sensitised and sensitised non‐allergic patients, potentially reducing the need for OFC [8, 213, 214, 215]. However, ca. 10% of patients exhibit basophil non‐responsiveness to FcεRI signalling [215, 216]. In such cases, MAT may serve as a valuable alternative. MCs can be differentiated from peripheral blood progenitors or alternatively immortalised human MC lines, such as Hox8 or LAD2, which can be passively sensitised using plasma [217, 218, 219, 220, 221]. Upon stimulation with allergens, MC degranulation is assessed using CD107a, CD63 or quantification of mediators released [222]. Even though MC lines can be passively sensitised and therefore do not reflect the patient's individual humoral environment, their use is facilitated by the possibility to store and work with frozen cells. Similarly, basophils can be passively sensitised by stripping to remove bound IgE using acidic buffers, followed by incubation with patients' serum [223, 224]. Both BAT and MAT are important tools for diagnosis and for studying allergies and tolerance development and have shown better performance than histamine release assays [225]. They can mimic the sequence of events in an in vivo situation since the upregulation of activation markers is delayed ex vivo [226].

Already in 1999, segmental mucosal challenges with food were performed and demonstrated that intraluminal administration of allergens leads to the release of mediators, such as tryptase, PDG_2_ and histamine within the first 15 min of an allergic reaction [227]. Biopsy‐based models offer insights into the complex interplay between immune and structural cells during allergic reactions. For example, GI biopsies were used to characterise B‐cells in peanut‐allergic individuals; here, it was shown that IgE‐expressing B‐cells were enriched in the stomach and duodenum, and allergic patients have a common pattern of immunoglobulin genetic rearrangements [103]. Similarly, during colonoscopic allergen provocations, MC degranulation could be demonstrated [228]. Despite being powerful tools to analyse local effects, biopsy‐based models do not allow studying the physiological sequence of events during an allergic reaction. Hung et al. recently showed that precision‐cut intestinal slices (PCIS) can be sensitised with plasma from allergic patients. These PCIS showed significantly increased muscle contractions and MC mediator release upon stimulation with the respective allergen [229]. Similar models using precision‐cut lung slices (PCLS) have been employed to study allergic airway inflammation and hyper‐responsiveness, as sensitised and allergen‐stimulated PCLS show immediate airway contractions [230, 231, 232, 233, 234].

### 4.2 Biomarkers of Acute IgE‐Mediated Food Allergy in Animal Models

Murine models have been extensively used to examine mechanisms of anaphylaxis using different sensitisation and challenge protocols (Figure 5) [235]. While most models utilise systemic sensitisation (e.g., intraperitoneal injection) with an adjuvant, epicutaneous sensitisation has been shown to elicit potent anaphylactic responses, including interesting age‐related effects on DC migration, Th2 polarisation, IgE production and body temperature decline, which is potentially mediated by IL‐33 [236]. The choice of murine strain is important as recently it was demonstrated that sensitisation to peanuts was successful in C3H mice and triggered production of allergen‐specific antibodies, cytokines and anaphylaxis, but much lower or no hypersensitivity reactions were observed to peanuts in BALB/c and C57BL/6 mice [237].

Alternatively, mucosal adjuvants can be used during oral sensitisation to promote activation of antigen‐presenting cells and T‐cell differentiation towards Th2. The most frequently used adjuvant is cholera toxin in combination with oral allergen application, leading to robust antigen‐specific IgE production and FA. This model was first described using ovalbumin and hen's egg lysate and was later optimised and adjusted to several different allergens and extracts. Despite many advantages of this model, including fast and strong sensitisation, the obtained results have to be interpreted carefully because of the potential immune‐activating properties of the adjuvant itself [238, 239, 240].

Humanised models using non‐obese diabetic severe combined immunodeficient common gamma chain‐deficient stem cell factor (huNSG) mice can be engrafted with functional human T and B lymphocytes, as well as human MCs. Challenge with peanuts resulted in MC‐mediated systemic anaphylaxis, while anti‐IgE treatment ablated this anaphylactic response [241]. In addition, colonisation of mice with a human microbiota influences the severity of anaphylaxis whereby the microbiota from healthy infants protects against anaphylaxis and the microbiota from allergic infants promotes anaphylaxis in recipient animals [242, 243]. It is thought that differences in taxa composition and microbial metabolic output (e.g., SCFAs) significantly impact MC responsiveness and Th2 sensitisation [244, 245, 246].

## 5 Future Challenges & Conclusions

Despite advances in understanding the acute immune response during FA reactions, several challenges remain. In vivo insights into the reaction cascade are currently limited, largely due to the difficulty in obtaining sufficient tissue samples during reactions. Consequently, there is a pressing need for more OFC studies that comprehensively analyse immune markers to better synthesise and interpret these data. While predicting reaction thresholds during OFCs appears increasingly feasible, accurately forecasting reaction severity remains elusive. Moreover, much of the current research focuses on the involvement of the intestinal immune system, with comparatively less attention given to the role of the oral mucosa in initiating and propagating allergic responses. In parallel with improving diagnostic and predictive tools, the development of novel therapeutic strategies is critical. For example, BTK, JAK1, as well as Siglec‐8 in MCs or basophils have emerged as promising molecular targets, whose inhibition may modulate acute allergic responses. Moving forward, a critical challenge lies in translating these emerging immunological findings and novel therapies into practical clinical guidelines that can improve patient management and risk stratification. Addressing these gaps will be essential for advancing personalised approaches to FA diagnosis, prognosis and treatment.

## Author Contributions

N.A.N., D.S., V.H., L.O. and E.F.K. wrote the manuscript. L.O., E.F.K. and A.F.S. edited the manuscript and provided critical input. A.K. and T.E. supervised writing and carefully edited the manuscript.

## Funding

This work was supported by the Luxembourg National Research Fund on the WEAVE program (grant C23/BM/18096599/IFAM), the Austrian Science Fund on the WEAVE program (grant 10.55776/I6897) and the Lower Austrian Research Fund, which supported the Danube Allergy Research Cluster.

## Conflicts of Interest

N.A.N., D.S., V.H., E.F.K. and A.K. declare that they have no conflicts of interest regarding this review. L.O. reports grants from Chiesi, Reckitt and Fonterra, and participation in a speaker bureau for Nestle, Yakult, Reckitt and Abbott. A.F.S. reports grants from the Medical Research Council (MR/M008517/1; MC/PC/18052; MR/T032081/1), Food Allergy Research and Education (FARE), the Immune Tolerance Network/National Institute of Allergy and Infectious Diseases (NIAID, NIH), Asthma UK (AUK‐BC‐2015‐01), BBSRC, Rosetrees Trust, and the NIHR through the Biomedical Research Centre (BRC) award to Guy's and St Thomas' NHS Foundation Trust; personal fees from Thermo Scientific, Novartis, Allergy Therapeutics, Buhlmann, Nestle, IgGenix, DBV Technologies, Sanofi, Regeneron, as well as research support from Thermo Fisher Scientific and IgGenix through a collaboration agreement with King's College London. T.E. reports to act as local PI for company‐sponsored trials by DBV Therapeutics, Greer Stallergens and subinvestigator for Regeneron and ALK‐Abelló. He is a coinvestigator or scientific lead in three investigator‐initiated oral immunotherapy trials supported by the SickKids Food Allergy and Anaphylaxis Program and serves as an associate editor for Allergy. He/his lab received unconditional/in‐kind contributions from Macro Array Diagnostics and an unrestricted grant from ALK‐Abelló. He holds advisory board roles for ALK‐Abelló, VAMED, Nutricia/Danone, Allergy Therapeutics and Aimmune. T.E. reports lecture fees from Novartis, ThermoFisher, Nutricia/Danone, Aimmune, ALK‐Abelló, MADX and Greer Stallergenes.

## Data Availability

Data sharing is not applicable to this article as no datasets were generated or analysed during the current study.

## References

[1] R. S. Gupta , C. M. Warren , B. M. Smith , et al., “Prevalence and Severity of Food Allergies Among US Adults,” JAMA Network Open 2, no. 1 (2019): e185630, https://jamanetwork.com/journals/jamanetworkopen/fullarticle/2720064. 30646188 10.1001/jamanetworkopen.2018.5630 PMC6324316

[2] R. S. Gupta , C. M. Warren , B. M. Smith , et al., “The Public Health Impact of Parent‐Reported Childhood Food Allergies in the United States,” Pediatrics 142 (2018): e20181235, 10.1542/peds.2018-1235. 30455345 PMC6317772

[3] R. J. Rona , T. Keil , C. Summers , et al., “The Prevalence of Food Allergy: A Meta‐Analysis,” Journal of Allergy and Clinical Immunology 120, no. 3 (2007): 638–646. 17628647 10.1016/j.jaci.2007.05.026

[4] J. W. Hahn , K. Lee , J. I. Shin , et al., “Global Incidence and Prevalence of Eosinophilic Esophagitis, 1976–2022: A Systematic Review and Meta‐Analysis,” Clinical Gastroenterology and Hepatology 21, no. 13 (2023): 3270–3284.e77. 37331411 10.1016/j.cgh.2023.06.005

[5] C. M. Warren , J. Jiang , and R. S. Gupta , “Epidemiology and Burden of Food Allergy,” Current Allergy and Asthma Reports 20, no. 2 (2020): 1–9, 10.1007/s11882-020-0898-7. 32067114 PMC7883751

[6] M. Al‐Iede , L. Sarhan , M. A. Alshrouf , and Y. Said , “Perspectives on Non‐IgE‐Mediated Gastrointestinal Food Allergy in Pediatrics: A Review of Current Evidence and Guidelines,” Journal of Asthma and Allergy 16 (2023): 279–291, https://pubmed.ncbi.nlm.nih.gov/36942164/. 36942164 10.2147/JAA.S284825 PMC10024490

[7] H. Yoshisue , Y. Homma , C. Ito , and M. Ebisawa , “Prevalence of Food Allergy Increased 1.7 Times in the Past 10 Years Among Japanese Patients Below 6 Years of Age,” Pediatric Allergy and Immunology 35, no. 7 (2024): e14192, 10.1111/pai.14192. 39007449

[8] A. F. Santos , C. Riggioni , I. Agache , et al., “EAACI Guidelines on the Diagnosis of IgE‐Mediated Food Allergy,” Allergy 78, no. 12 (2023): 3057–3076, 10.1111/all.15902. 37815205

[9] H. A. Sampson , S. Aceves , S. A. Bock , et al., “Food Allergy: A Practice Parameter Update‐2014,” Journal of Allergy and Clinical Immunology 134, no. 5 (2014): 1016–1025.e43, https://pubmed.ncbi.nlm.nih.gov/25174862/. 25174862 10.1016/j.jaci.2014.05.013

[10] L. B. Grabenhenrich , S. Dölle , A. Moneret‐Vautrin , et al., “Anaphylaxis in Children and Adolescents: The European Anaphylaxis Registry,” Journal of Allergy and Clinical Immunology 137, no. 4 (2016): 1128–1137.e1, https://pubmed.ncbi.nlm.nih.gov/26806049/. 26806049 10.1016/j.jaci.2015.11.015

[11] J. Upton and T. Eiwegger , “How Low Should we Go,” International Archives of Allergy and Immunology 168, no. 3 (2015): 147–149, https://pubmed.ncbi.nlm.nih.gov/26788722/. 26788722 10.1159/000443764

[12] P. J. Turner , D. E. Campbell , M. S. Motosue , and R. L. Campbell , “Global Trends in Anaphylaxis Epidemiology and Clinical Implications,” Journal of Allergy and Clinical Immunology: In Practice 8, no. 4 (2020): 1169, https://pmc.ncbi.nlm.nih.gov/articles/PMC7152797/. 31786255 10.1016/j.jaip.2019.11.027 PMC7152797

[13] E. Hochstadter , A. Clarke , S. De Schryver , et al., “Increasing Visits for Anaphylaxis and the Benefits of Early Epinephrine Administration: A 4‐Year Study at a Pediatric Emergency Department in Montreal, Canada,” Journal of Allergy and Clinical Immunology 137, no. 6 (2016): 1888–1890.e4, https://pubmed.ncbi.nlm.nih.gov/27106202/. 27106202 10.1016/j.jaci.2016.02.016

[14] F. E. R. Simons , M. Ebisawa , M. Sanchez‐Borges , et al., “2015 Update of the Evidence Base: World Allergy Organization Anaphylaxis Guidelines,” World Allergy Organization Journal 8, no. 1 (2015): 32, https://pubmed.ncbi.nlm.nih.gov/26525001/. 26525001 10.1186/s40413-015-0080-1 PMC4625730

[15] M. Worm , A. Moneret‐Vautrin , K. Scherer , et al., “First European Data From the Network of Severe Allergic Reactions (NORA),” Allergy 69, no. 10 (2014): 1397–1404, https://pubmed.ncbi.nlm.nih.gov/24989080/. 24989080 10.1111/all.12475

[16] M. Ponce , S. C. Diesner , Z. Szépfalusi , and T. Eiwegger , “Markers of Tolerance Development to Food Allergens,” Allergy 71, no. 10 (2016): 1393–1404, https://pubmed.ncbi.nlm.nih.gov/27286276/. 27286276 10.1111/all.12953

[17] E. G. A. Iglesia , M. Kwan , Y. V. Virkud , and O. I. Iweala , “Management of Food Allergies and Food‐Related Anaphylaxis,” JAMA 331, no. 6 (2024): 510–521, https://pubmed.ncbi.nlm.nih.gov/38349368/. 38349368 10.1001/jama.2023.26857 PMC11060332

[18] R. Czolk , J. Klueber , M. Sørensen , et al., “IgE‐Mediated Peanut Allergy: Current and Novel Predictive Biomarkers for Clinical Phenotypes Using Multi‐Omics Approaches,” Frontiers in Immunology 11 (2021): 594350, https://pubmed.ncbi.nlm.nih.gov/33584660/. 33584660 10.3389/fimmu.2020.594350 PMC7876438

[19] A. Muraro , T. Werfel , K. Hoffmann‐Sommergruber , et al., “EAACI Food Allergy and Anaphylaxis Guidelines: Diagnosis and Management of Food Allergy,” Allergy 69, no. 8 (2014): 1008–1025, https://pubmed.ncbi.nlm.nih.gov/24909706/. 24909706 10.1111/all.12429

[20] S. U. Patil , S. Bunyavanich , and M. C. Berin , “Emerging Food Allergy Biomarkers,” Journal of Allergy and Clinical Immunology: In Practice 8, no. 8 (2020): 2516, https://pmc.ncbi.nlm.nih.gov/articles/PMC7479640/. 32888527 10.1016/j.jaip.2020.04.054 PMC7479640

[21] K. W. Chong , M. Ruiz‐Garcia , N. Patel , R. J. Boyle , and P. J. Turner , “Reaction Phenotypes in IgE‐Mediated Food Allergy and Anaphylaxis,” Annals of Allergy, Asthma & Immunology 124, no. 5 (2020): 473–478. 10.1016/j.anai.2019.12.023 PMC7251627 31923546

[22] M. Malucelli , R. Farias , R. G. Mello , and C. Prando , “Biomarkers Associated With Persistence and Severity of IgE‐Mediated Food Allergies: A Systematic Review,” Jornal de Pediatria 99, no. 4 (2023): 315, https://pmc.ncbi.nlm.nih.gov/articles/PMC10373149/. 36977497 10.1016/j.jped.2023.02.004 PMC10373149

[23] P. J. Turner , S. Arasi , B. Ballmer‐Weber , et al., “Risk Factors for Severe Reactions in Food Allergy: Rapid Evidence Review With Meta‐Analysis,” Allergy 77, no. 9 (2022): 2634–2652. 35441718 10.1111/all.15318 PMC9544052

[24] M. R. Datema , S. A. Lyons , M. Fernández‐Rivas , et al., “Estimating the Risk of Severe Peanut Allergy Using Clinical Background and IgE Sensitization Profiles,” Frontiers in Allergy 2 (2021): 670789. 35386994 10.3389/falgy.2021.670789 PMC8974676

[25] B. Sprinzl , G. Greiner , G. Uyanik , et al., “Genetic Regulation of Tryptase Production and Clinical Impact: Hereditary Alpha Tryptasemia, Mastocytosis and Beyond,” International Journal of Molecular Sciences 22 (2021): 2458, https://www.mdpi.com/1422‐0067/22/5/2458/htm. 33671092 10.3390/ijms22052458 PMC7957558

[26] K. T. Luskin , A. A. White , and J. J. Lyons , “The Genetic Basis and Clinical Impact of Hereditary Alpha‐Tryptasemia,” Journal of Allergy and Clinical Immunology. In Practice 9, no. 6 (2021): 2235–2242. 33744473 10.1016/j.jaip.2021.03.005

[27] Y. Chantran , H. Renaudin , M. Arock , T. Guiddir , and A. Nemni , “Case Report: A Family History of Peanut Allergy and Hereditary Alpha‐Tryptasemia,” Frontiers in Allergy 4 (2023): 1322117. 38327735 10.3389/falgy.2023.1322117 PMC10848325

[28] J. J. Lyons , J. Chovanec , M. P. O'Connell , et al., “Heritable Risk for Severe Anaphylaxis Associated With Increased α‐Tryptase–Encoding Germline Copy Number at TPSAB1,” Journal of Allergy and Clinical Immunology 147, no. 2 (2021): 622–632. 32717252 10.1016/j.jaci.2020.06.035

[29] A. H. Benhamou , S. A. Zamora , and P. A. Eigenmann , “Correlation Between Specific Immunoglobulin E Levels and the Severity of Reactions in Egg Allergic Patients,” Pediatric Allergy and Immunology 19, no. 2 (2008): 173–179, https://pubmed.ncbi.nlm.nih.gov/18257905/. 18257905 10.1111/j.1399-3038.2007.00602.x

[30] P. J. Turner and A. Custovic , “Life‐Threatening Anaphylaxis to Peanut—Impossible to Predict?,” Journal of Allergy and Clinical Immunology 149, no. 3 (2022): 1128–1129. 35000773 10.1016/j.jaci.2021.12.767

[31] A. F. Santos , E. Evans , C. O'Rourke , H. T. Bahnson , and G. Lack , “Reply,” Journal of Allergy and Clinical Immunology 149, no. 3 (2022): 1129–1131. 35000772 10.1016/j.jaci.2021.12.766

[32] R. L. Peters , K. J. Allen , S. C. Dharmage , et al., “Skin Prick Test Responses and Allergen‐Specific IgE Levels as Predictors of Peanut, Egg, and Sesame Allergy in Infants,” Journal of Allergy and Clinical Immunology 132, no. 4 (2013): 874–880, https://pubmed.ncbi.nlm.nih.gov/23891354/. 23891354 10.1016/j.jaci.2013.05.038

[33] C. G. Uasuf , D. Villalta , M. E. Conte , et al., “Different Co‐Sensitizations Could Determine Different Risk Assessment in Peach Allergy? Evaluation of an Anaphylactic Biomarker in Pru p 3 Positive Patients,” Clinical and Molecular Allergy 13, no. 1 (2015): 30. 26633941 10.1186/s12948-015-0035-7 PMC4667484

[34] E. González‐Mancebo , D. González‐de‐Olano , M. J. Trujillo , et al., “Prevalence of Sensitization to Lipid Transfer Proteins and Profilins in a Population of 430 Patients in the South of Madrid,” Journal of Investigational Allergology & Clinical Immunology 21, no. 4 (2011): 278–282. 21721373

[35] F. El‐Khouly , S. A. Lewis , L. Pons , A. W. Burks , and J. O. Hourihane , “IgG and IgE Avidity Characteristics of Peanut Allergic Individuals,” Pediatric Allergy and Immunology 18, no. 7 (2007): 607–613. 18001431 10.1111/j.1399-3038.2007.00542.x

[36] O. Hemmings , U. Niazi , M. Kwok , L. K. James , G. Lack , and A. F. Santos , “Peanut Diversity and Specific Activity Are the Dominant IgE Characteristics for Effector Cell Activation in Children,” Journal of Allergy and Clinical Immunology 148, no. 2 (2021): 495–505.e14. 33675817 10.1016/j.jaci.2021.02.029 PMC8340728

[37] T. Eiwegger , L. Hung , K. E. San Diego , L. O'Mahony , and J. Upton , “Recent Developments and Highlights in Food Allergy,” Allergy 74, no. 12 (2019): 2355–2367, 10.1111/all.14082. 31593325

[38] M. F. Bachmann , P. S. Krenger , M. O. Mohsen , et al., “On the Role of Antibody Affinity and Avidity in the IgE‐Mediated Allergic Response,” Allergy 80, no. 1 (2025): 37–46, 10.1111/all.16248. 39189064 PMC11724228

[39] S. A. Smith , Y. W. Khan , R. A. Shrem , et al., “Antigenic Determinants Underlying IgE‐Mediated Anaphylaxis to Peanut,” Journal of Allergy and Clinical Immunology 155 (2025): 1595–1606.e10. 39814145 10.1016/j.jaci.2024.12.1094

[40] K. Plattner , M. F. Bachmann , and M. Vogel , “On the Complexity of IgE: The Role of Structural Flexibility and Glycosylation for Binding Its Receptors,” Frontiers in Allergy 4 (2023): 1117611. 37056355 10.3389/falgy.2023.1117611 PMC10089267

[41] K. T. Shade , M. E. Conroy , and R. M. Anthony , “IgE Glycosylation in Health and Disease,” Current Topics in Microbiology and Immunology 423 (2019): 77, https://pmc.ncbi.nlm.nih.gov/articles/PMC6750212/. 30820668 10.1007/82_2019_151 PMC6750212

[42] K. Plattner , Z. Gharailoo , S. Zinkhan , P. Engeroff , M. F. Bachmann , and M. Vogel , “IgE Glycans Promote Anti‐IgE IgG Autoantibodies That Facilitate IgE Serum Clearance via Fc Receptors,” Frontiers in Immunology 13 (2022): 1069100. 36544773 10.3389/fimmu.2022.1069100 PMC9761184

[43] T. Nakamura and T. Murata , “Regulation of Vascular Permeability in Anaphylaxis,” British Journal of Pharmacology 175, no. 13 (2018): 2538, https://pmc.ncbi.nlm.nih.gov/articles/PMC6003654/. 29671869 10.1111/bph.14332 PMC6003654

[44] S. G. A. Brown , “Cardiovascular Aspects of Anaphylaxis: Implications for Treatment and Diagnosis,” Current Opinion in Allergy and Clinical Immunology 5, no. 4 (2005): 359–364, https://pubmed.ncbi.nlm.nih.gov/15985820/. 15985820 10.1097/01.all.0000174158.78626.35

[45] S. M. T. Nguyen , C. P. Rupprecht , A. Haque , D. Pattanaik , J. Yusin , and G. Krishnaswamy , “Mechanisms Governing Anaphylaxis: Inflammatory Cells, Mediators, Endothelial Gap Junctions and Beyond,” International Journal of Molecular Sciences 22, no. 15 (2021): 7785, https://www.mdpi.com/1422‐0067/22/15/7785/htm. 34360549 10.3390/ijms22157785 PMC8346007

[46] M. Krawiec , S. Radulovic , R. Foong , et al., “Diagnostic Utility of Allergy Tests to Predict Baked Egg and Lightly Cooked Egg Allergies Compared to Double‐Blind Placebo‐Controlled Food Challenges,” Allergy 78, no. 9 (2023): 2510–2522. 37417650 10.1111/all.15797 PMC10790315

[47] S. Radulovic , R. Foong , I. Bartha , et al., “Basophil Activation Test as Predictor of Severity and Threshold of Allergic Reactions to Egg,” Allergy 79, no. 2 (2024): 419–431. 37680143 10.1111/all.15875 PMC10952485

[48] A. F. Santos , G. Du Toit , A. Douiri , et al., “Distinct Parameters of the Basophil Activation Test Reflect the Severity and Threshold of Allergic Reactions to Peanut,” Journal of Allergy and Clinical Immunology 135, no. 1 (2015): 179–186. 25567046 10.1016/j.jaci.2014.09.001 PMC4282725

[49] R. S. Chinthrajah , N. Purington , S. Andorf , et al., “Development of a Tool Predicting Severity of Allergic Reaction During Peanut Challenge,” Annals of Allergy, Asthma & Immunology 121, no. 1 (2018): 69–76.e2. 10.1016/j.anai.2018.04.020 PMC6026554 29709643

[50] A. F. Santos , G. Du Toit , C. O'Rourke , et al., “Biomarkers of Severity and Threshold of Allergic Reactions During Oral Peanut Challenges,” Journal of Allergy and Clinical Immunology 146, no. 2 (2020): 344–355. 32311390 10.1016/j.jaci.2020.03.035 PMC7417812

[51] N. Cottel , S. Saf , M. Bourgoin‐Heck , et al., “Two Different Composite Markers Predict Severity and Threshold Dose in Peanut Allergy,” Journal of Allergy and Clinical Immunology. In Practice 9, no. 1 (2021): 275–282.e1. 33038591 10.1016/j.jaip.2020.09.043

[52] O. Zenarruzabeitia , J. Vitallé , I. Terrén , et al., “CD300c Costimulates IgE‐Mediated Basophil Activation, and Its Expression Is Increased in Patients With Cow's Milk Allergy,” Journal of Allergy and Clinical Immunology 143, no. 2 (2019): 700–711.e5. 29906528 10.1016/j.jaci.2018.05.022

[53] P. Vadas , M. Gold , B. Perelman , et al., “Platelet‐Activating Factor, PAF Acetylhydrolase, and Severe Anaphylaxis,” New England Journal of Medicine 358, no. 1 (2008): 28–35, 10.1056/NEJMoa070030. 18172172

[54] J. E. M. Upton , J. A. Hoang , M. Leon‐Ponte , et al., “Platelet‐Activating Factor Acetylhydrolase Is a Biomarker of Severe Anaphylaxis in Children,” Allergy 77, no. 9 (2022): 2665–2676, 10.1111/all.15308. 35396721

[55] K. T. C. Shade , M. E. Conroy , N. Washburn , et al., “Sialylation of Immunoglobulin E Is a Determinant of Allergic Pathogenicity,” Nature 582, no. 7811 (2020): 265–270. 32499653 10.1038/s41586-020-2311-z PMC7386252

[56] L. Dühring , J. Petry , G. Lilienthal , et al., “Sialylation of IgE Reduces FcεRIα Interaction and Mast Cell and Basophil Activation In Vitro and Increases IgE Half‐Life In Vivo,” Allergy 78, no. 8 (2023): 2301–2305. 36724158 10.1111/all.15665

[57] H. e. Ho , Z. Arditi , L. Radigan , et al., “Saliva Antibody Profiles Are Associated With Reaction Threshold and Severity of Peanut Allergic Reactions,” Journal of Allergy and Clinical Immunology 154, no. 3 (2024): 690–697.e4. 38821318 10.1016/j.jaci.2024.05.020 PMC11380589

[58] A. Lang , S. Kubala , M. C. Grieco , et al., “Severe Food Allergy Reactions Are Associated With α‐Tryptase,” Journal of Allergy and Clinical Immunology 152, no. 4 (2023): 933–939, https://pubmed.ncbi.nlm.nih.gov/37558059/. 37558059 10.1016/j.jaci.2023.07.014 PMC10592152

[59] D. Loli‐Ausejo , M. F. Gonzalez‐Matamala , D. Martí , et al., “Basal Serum Tryptase Outperforms Hereditary Alpha Tryptasemia as a Biomarker of Food Allergy Severity,” Allergy 80 (2025): 2376–2378. 40503609 10.1111/all.16623 PMC12368740

[60] R. Khalaf , C. Prosty , W. Davalan , E. Abrams , M. Kaouache , and M. Ben‐Shoshan , “Diagnostic Utility of Biomarkers in Anaphylaxis: A Systematic Review and Meta‐Analysis,” Journal of Allergy and Clinical Immunology. In Practice 13, no. 6 (2025): 1342–1349.e12. 40239922 10.1016/j.jaip.2025.04.008

[61] R. Y. Lin , L. B. Schwartz , A. Curry , et al., “Histamine and Tryptase Levels in Patients With Acute Allergic Reactions: An Emergency Department–Based Study,” Journal of Allergy and Clinical Immunology 106, no. 1 (2000): 65–71. 10887307 10.1067/mai.2000.107600

[62] Y.‐Q. Zhu , D.‐Q. Wang , B. Liu , et al., “Wheat‐Dependent Exercise‐Induced Anaphylaxis in Chinese People: A Clinical Research on 33 Cases With Antigenic Analysis of Wheat Proteins,” Clinical and Experimental Dermatology 45, no. 1 (2020): 56–62. 31267575 10.1111/ced.14035

[63] S. Inagaki , S. Maeda , M. Narita , et al., “Urinary PGDM, a Prostaglandin D2 Metabolite, Is a Novel Biomarker for Objectively Detecting Allergic Reactions of Food Allergy,” Journal of Allergy and Clinical Immunology 142, no. 5 (2018): 1634–1636.e10. 29981807 10.1016/j.jaci.2018.06.032

[64] K. Umezawa , N. Nagata , S. Kabashima , et al., “Urinary Prostaglandin Metabolites as Potential Biomarkers for Differentiating IgE‐Mediated Food Allergy and Food Protein‐Induced Enterocolitis Syndrome,” Allergy 80 (2025): 2890–2893. 40371994 10.1111/all.16589

[65] S. F. Stone , C. Cotterell , G. K. Isbister , A. Holdgate , and S. G. A. Brown , “Elevated Serum Cytokines During Human Anaphylaxis: Identification of Potential Mediators of Acute Allergic Reactions,” Journal of Allergy and Clinical Immunology 124, no. 4 (2009): 786–792.e4. 19767073 10.1016/j.jaci.2009.07.055

[66] A. Francis , E. Bosio , S. F. Stone , et al., “Markers Involved in Innate Immunity and Neutrophil Activation Are Elevated During Acute Human Anaphylaxis: Validation of a Microarray Study,” Journal of Innate Immunity 11, no. 1 (2019): 63–73. 30189430 10.1159/000492301 PMC6738247

[67] M. Kara , O. F. Beser , D. Konukoglu , et al., “The Utility of TNF‐α, IL‐6 and IL‐10 in the Diagnosis and/or Follow‐Up Food Allergy,” Allergologia et Immunopathologia 48, no. 1 (2020): 48–55. 31732222 10.1016/j.aller.2019.04.011

[68] B. Perelman , A. Adil , and P. Vadas , “Relationship Between Platelet Activating Factor Acetylhydrolase Activity and Apolipoprotein B Levels in Patients With Peanut Allergy,” Allergy, Asthma and Clinical Immunology 10, no. 1 (2014): 1–4, 10.1186/1710-1492-10-20. PMC4012516 24808915

[69] P. Gill , N. L. Jindal , A. Jagdis , and P. Vadas , “Platelets in the Immune Response: Revisiting Platelet‐Activating Factor in Anaphylaxis,” Journal of Allergy and Clinical Immunology 135, no. 6 (2015): 1424–1432, https://pubmed.ncbi.nlm.nih.gov/26051949/. 26051949 10.1016/j.jaci.2015.04.019

[70] P. Röntynen , K. Kukkonen , T. Savinko , and M. J. Mäkelä , “Interaction of Mediators and Effector Cells in Cashew Nut‐Induced Anaphylaxis,” Annals of Allergy, Asthma & Immunology 131, no. 2 (2023): 239–252.e6, https://pubmed.ncbi.nlm.nih.gov/37098406/. 10.1016/j.anai.2023.04.014 37098406

[71] K. L. Piwowarek , A. Rzeszotarska , J. L. Korsak , A. Juszkiewicz , A. Chcialowski , and J. Kruszewski , “Clinical Significance of Plasma PAF Acetylhydrolase Activity Measurements as a Biomarker of Anaphylaxis: Cross‐Sectional Study,” PLoS One 16, no. 8 (2021): e0256168, https://pmc.ncbi.nlm.nih.gov/articles/PMC8362976/. 34388201 10.1371/journal.pone.0256168 PMC8362976

[72] J. E. M. Upton , E. Grunebaum , G. Sussman , and P. Vadas , “Platelet Activating Factor (PAF): A Mediator of Inflammation,” BioFactors 48, no. 6 (2022): 1189–1202, https://pubmed.ncbi.nlm.nih.gov/36029481/. 36029481 10.1002/biof.1883

[73] Y. Zhang , F. Collier , G. Naselli , et al., “Cord Blood Monocyte–Derived Inflammatory Cytokines Suppress IL‐2 and Induce Nonclassic “T_H_ 2‐Type” Immunity Associated With Development of Food Allergy,” Science Translational Medicine 8, no. 321 (2016): 321ra8. 10.1126/scitranslmed.aad4322 26764159

[74] M. K. Tulic , M. Hodder , A. Forsberg , et al., “Differences in Innate Immune Function Between Allergic and Nonallergic Children: New Insights Into Immune Ontogeny,” Journal of Allergy and Clinical Immunology 127, no. 2 (2011): 470–478.e1. 21093030 10.1016/j.jaci.2010.09.020

[75] M. R. Neeland , J. J. Koplin , T. D. Dang , et al., “Early Life Innate Immune Signatures of Persistent Food Allergy,” Journal of Allergy and Clinical Immunology 142, no. 3 (2018): 857–864.e3. 29154959 10.1016/j.jaci.2017.10.024

[76] A. Dang , S. Logsdon , and S. P. Hogan , “Investigating Innate Immune Mechanisms in Early‐Life Development and Outcomes of Food Allergy,” Journal of Allergy and Clinical Immunology 142, no. 3 (2018): 790–792. 30096392 10.1016/j.jaci.2018.08.001 PMC8215521

[77] K. Oishi , N. Nakano , M. Ota , et al., “MHC Class II‐Expressing Mucosal Mast Cells Promote Intestinal Mast Cell Hyperplasia in a Mouse Model of Food Allergy,” Allergy 80, no. 8 (2025): 2297–2309, https://pubmed.ncbi.nlm.nih.gov/39868907/. 39868907 10.1111/all.16477

[78] L. Ollé , L. García‐García , M. Ruano‐Zaragoza , et al., “Mast Cell Activation Profile and TFH13 Detection Discriminate Between Food Anaphylaxis and Sensitization,” Journal of Investigational Allergology & Clinical Immunology 35, no. 3 (2025): 203–215. 38381397 10.18176/jiaci.0995

[79] S. Benedé and M. C. Berin , “Mast Cell Heterogeneity Underlies Different Manifestations of Food Allergy in Mice,” PLoS One 13, no. 1 (2018): e0190453, https://pmc.ncbi.nlm.nih.gov/articles/PMC5784907/. 29370173 10.1371/journal.pone.0190453 PMC5784907

[80] N. Nakano and J. Kitaura , “Mucosal Mast Cells as Key Effector Cells in Food Allergies,” Cells 11, no. 3 (2022): 329, https://pmc.ncbi.nlm.nih.gov/articles/PMC8834119/. 35159139 10.3390/cells11030329 PMC8834119

[81] J. Calise , N. Garabatos , V. Bajzik , et al., “Optimal Human Pathogenic TH2 Cell Effector Function Requires Local Epithelial Cytokine Signaling,” Journal of Allergy and Clinical Immunology 148, no. 3 (2021): 867–875.e4, https://pubmed.ncbi.nlm.nih.gov/33662368/. 33662368 10.1016/j.jaci.2021.02.019 PMC8408285

[82] E. Wambre , V. Bajzik , J. H. DeLong , et al., “A Phenotypically and Functionally Distinct Human TH2 Cell Subpopulation Is Associated With Allergic Disorders,” Science Translational Medicine 9, no. 401 (2017): eaam9171. 28768806 10.1126/scitranslmed.aam9171 PMC5987220

[83] M. C. Berin , A. Grishin , M. Masilamani , et al., “Egg‐Specific IgE and Basophil Activation but Not Egg‐Specific T‐Cell Counts Correlate With Phenotypes of Clinical Egg Allergy,” Journal of Allergy and Clinical Immunology 142, no. 1 (2018): 149–158.e8, https://pubmed.ncbi.nlm.nih.gov/29518422/. 29518422 10.1016/j.jaci.2018.01.044 PMC6282170

[84] D. Chiang , X. Chen , S. M. Jones , et al., “Single‐Cell Profiling of Peanut‐Responsive T Cells in Patients With Peanut Allergy Reveals Heterogeneous Effector TH2 Subsets,” Journal of Allergy and Clinical Immunology 141, no. 6 (2018): 2107–2120, https://pubmed.ncbi.nlm.nih.gov/29408715/. 29408715 10.1016/j.jaci.2017.11.060 PMC5994177

[85] M. C. Berin , C. Agashe , A. W. Burks , et al., “Allergen‐Specific T Cells and Clinical Features of Food Allergy: Lessons From CoFAR Immunotherapy Cohorts,” Journal of Allergy and Clinical Immunology 149, no. 4 (2022): 1373–1382.e12, https://pubmed.ncbi.nlm.nih.gov/34653515/. 34653515 10.1016/j.jaci.2021.09.029 PMC8995337

[86] V. Bajzik , H. A. DeBerg , N. Garabatos , et al., “Oral Desensitization Therapy for Peanut Allergy Induces Dynamic Changes in Peanut‐Specific Immune Responses,” Allergy 77, no. 8 (2022): 2534–2548. 35266148 10.1111/all.15276 PMC9356972

[87] B. Upadhyaya , Y. Yin , B. J. Hill , D. C. Douek , and C. Prussin , “Hierarchical IL‐5 Expression Defines a Subpopulation of Highly Differentiated Human Th2 Cells,” Journal of Immunology 187, no. 6 (2011): 3111–3120. 10.4049/jimmunol.1101283 PMC3445433 21849680

[88] S. Luce , S. Chinthrajah , S. C. Lyu , K. C. Nadeau , and L. Mascarell , “Th2A and Th17 Cell Frequencies and Regulatory Markers as Follow‐Up Biomarker Candidates for Successful Multifood Oral Immunotherapy,” Allergy 75, no. 6 (2020): 1513, https://pmc.ncbi.nlm.nih.gov/articles/PMC8164780/. 31930521 10.1111/all.14180 PMC8164780

[89] M. R. Neeland , S. Andorf , T. D. Dang , et al., “Altered Immune Cell Profiles and Impaired CD4 T‐Cell Activation in Single and Multi‐Food Allergic Adolescents,” Clinical & Experimental Allergy 51, no. 5 (2021): 674–684, 10.1111/cea.13857. 33626189

[90] G. Gri , S. Piconese , B. Frossi , et al., “CD^4+^CD^25+^ Regulatory T Cells Suppress Mast Cell Degranulation and Allergic Responses Through OX40‐OX40L Interaction,” Immunity 29, no. 5 (2008): 771–781. 18993084 10.1016/j.immuni.2008.08.018 PMC2590499

[91] P. Bacher , F. Heinrich , U. Stervbo , et al., “Regulatory T Cell Specificity Directs Tolerance Versus Allergy Against Aeroantigens in Humans,” Cell 167, no. 4 (2016): 1067–1078.e16. 27773482 10.1016/j.cell.2016.09.050

[92] B. Monian , A. A. Tu , B. Ruiter , et al., “Peanut Oral Immunotherapy Differentially Suppresses Clonally Distinct Subsets of T Helper Cells,” Journal of Clinical Investigation 132, no. 2 (2022): e150634. 34813505 10.1172/JCI150634 PMC8759778

[93] A. Abdel‐Gadir , L. Schneider , A. Casini , et al., “Oral Immunotherapy With Omalizumab Reverses the Th2 Cell‐Like Programme of Regulatory T Cells and Restores Their Function,” Clinical and Experimental Allergy 48, no. 7 (2018): 825–836. 29700872 10.1111/cea.13161 PMC6021220

[94] X. Zhou , D. Dunham , S. B. Sindher , et al., “HLA‐DR+ Regulatory T Cells and IL‐10 Are Associated With Success or Failure of Desensitization Outcomes,” Allergy 80, no. 3 (2025): 762–774. 39291303 10.1111/all.16311 PMC11893263

[95] A. Kaushik , D. Dunham , X. Han , et al., “CD8+ T Cell Differentiation Status Correlates With the Feasibility of Sustained Unresponsiveness Following Oral Immunotherapy,” Nature Communications 13, no. 1 (2022): 6646. 10.1038/s41467-022-34222-8 PMC9636180 36333296

[96] W. Yu , X. Zhou , S. C. Lyu , M. M. Davis , and K. C. Nadeau , “Regulation of Peanut‐Specific CD8+ T Cells From Nonallergic Individuals,” Journal of Allergy and Clinical Immunology 147, no. 1 (2021): 385–387.e1. 32835695 10.1016/j.jaci.2020.07.032 PMC7855141

[97] J. W. Krempski , T. Kobayashi , K. Iijima , A. N. McKenzie , and H. Kita , “Group 2 Innate Lymphoid Cells Promote Development of T Follicular Helper Cells and Initiate Allergic Sensitization to Peanuts,” Journal of Immunology 204, no. 12 (2020): 3086–3096, https://pubmed.ncbi.nlm.nih.gov/32366582/. 10.4049/jimmunol.2000029 PMC7309436 32366582

[98] J. Ilangovan , J. F. Neves , and A. F. Santos , “Innate Lymphoid Cells in Immunoglobulin E‐Mediated Food Allergy,” Current Opinion in Allergy and Clinical Immunology 24, no. 5 (2024): 419–425. 39132724 10.1097/ACI.0000000000001018 PMC11356679

[99] J. M. Leyva‐Castillo , C. Galand , C. Kam , et al., “Mechanical Skin Injury Promotes Food Anaphylaxis by Driving Intestinal Mast Cell Expansion,” Immunity 50, no. 5 (2019): 1262–1275.e4. 31027995 10.1016/j.immuni.2019.03.023 PMC6531322

[100] V. V. Ganusov and R. J. De Boer , “Do Most Lymphocytes in Humans Really Reside in the Gut?,” Trends in Immunology 28, no. 12 (2007): 514–518. 17964854 10.1016/j.it.2007.08.009

[101] H. Herbrand , G. Bernhardt , R. Forster , and O. Pabst , “Dynamics and Function of Solitary Intestinal Lymphoid Tissue,” Critical Reviews in Immunology 28, no. 1 (2008): 1–13. 18298381 10.1615/critrevimmunol.v28.i1.10

[102] R. A. Hoh and S. D. Boyd , “Gut Mucosal Antibody Responses and Implications for Food Allergy,” Frontiers in Immunology 9 (2018): 2221. 30319658 10.3389/fimmu.2018.02221 PMC6170638

[103] R. A. Hoh , S. A. Joshi , J. Y. Lee , et al., “Origins and Clonal Convergence of Gastrointestinal IgE+ B Cells in Human Peanut Allergy,” Science Immunology 5, no. 45 (2020): eaay4209, 10.1126/sciimmunol.aay4209. 32139586 PMC7691169

[104] J. F. E. Koenig , N. P. H. Knudsen , A. Phelps , K. Bruton , I. Hoof , and G. Lund , “A Distinct Phenotype of Polarized Memory B Cell Holds IgE Memory,” (2023).

[105] M. Ota , K. B. Hoehn , W. Fernandes‐Braga , et al., “CD23+ IgG1+ Memory B Cells Are Poised to Switch to Pathogenic IgE Production in Food Allergy,” Science Translational Medicine 16, no. 733 (2024): eadi0673. 38324641 10.1126/scitranslmed.adi0673 PMC11008013

[106] A. Poole , Y. Song , H. Brown , P. H. Hart , and G. Zhang , “Cellular and Molecular Mechanisms of Vitamin D in Food Allergy,” Journal of Cellular and Molecular Medicine 22, no. 7 (2018): 3270, https://pmc.ncbi.nlm.nih.gov/articles/PMC6010899/. 29577619 10.1111/jcmm.13607 PMC6010899

[107] E. Sdona , S. Ekström , N. Andersson , et al., “Dietary Fibre in Relation to Asthma, Allergic Rhinitis and Sensitization From Childhood up to Adulthood,” Clinical and Translational Allergy 12, no. 8 (2022): e12188, https://pmc.ncbi.nlm.nih.gov/articles/PMC9382355/. 35990418 10.1002/clt2.12188 PMC9382355

[108] G. Feketea , M. Kostara , R. S. Bumbacea , E. Vassilopoulou , and S. Tsabouri , “Vitamin D and Omega‐3 (Fatty Acid) Supplementation in Pregnancy for the Primary Prevention of Food Allergy in Children‐Literature Review,” Children (Basel) 10, no. 3 (2023): 468, https://www.mdpi.com/2227‐9067/10/3/468/htm. 36980026 10.3390/children10030468 PMC10047068

[109] T. Matsui , K. Tanaka , H. Yamashita , et al., “Food Allergy Is Linked to Season of Birth, Sun Exposure, and Vitamin D Deficiency,” Allergology International 68, no. 2 (2019): 172–177. 30670337 10.1016/j.alit.2018.12.003

[110] Z. Arditi and S. Bunyavanich , “Commensal Collaborations: Food Allergy and the Microbiome,” Journal of Allergy and Clinical Immunology 152, no. 6 (2023): 1417–1419, https://www.jacionline.org/action/showFullText?pii=S009167492300982X. 37558058 10.1016/j.jaci.2023.08.001 PMC11402024

[111] L. Zhang , Y. Chun , H. e. Ho , et al., “Multiscale Study of the Oral and Gut Environments in Children With High‐ and Low‐Threshold Peanut Allergy,” Journal of Allergy and Clinical Immunology 150, no. 3 (2022): 714–720.e2. 35550149 10.1016/j.jaci.2022.04.026 PMC9463091

[112] L. Zhang , Y. Chun , G. Grishina , et al., “Oral and Gut Microbial Hubs Associated With Reaction Threshold Interact With Circulating Immune Factors in Peanut Allergy,” Allergy 80 (2025): 2800–2809. 39887792 10.1111/all.16481 PMC12310987

[113] E. Sánchez‐Martínez , L. E. Rondeau , M. Garrido‐Romero , B. da Barbosa Luz , D. A. Haas , and G. Yuen , “Microbial Metabolism of Food Allergens Determines the Severity of IgE‐Mediated Anaphylaxis,” (2025). 10.1016/j.chom.2026.02.013 41780533

[114] M. Sasaki , N. H. A. Suaini , J. Afghani , et al., “Systematic Review of the Association Between Short‐Chain Fatty Acids and Allergic Diseases,” Allergy 79, no. 7 (2024): 1789–1811, https://pubmed.ncbi.nlm.nih.gov/38391245/. 38391245 10.1111/all.16065

[115] L. Paparo , R. Nocerino , E. Ciaglia , et al., “Butyrate as a Bioactive Human Milk Protective Component Against Food Allergy,” Allergy 76, no. 5 (2021): 1398–1415, 10.1111/all.14625. 33043467 PMC8247419

[116] M. Nshanian , J. J. Gruber , B. S. Geller , et al., “Short‐Chain Fatty Acid Metabolites Propionate and Butyrate Are Unique Epigenetic Regulatory Elements Linking Diet, Metabolism and Gene Expression,” Nature Metabolism 7, no. 1 (2025): 196–211, https://www.nature.com/articles/s42255‐024‐01191‐9. 10.1038/s42255-024-01191-9 PMC11774759 39789354

[117] A. S. R. Ballegaard and K. L. Bøgh , “Intestinal Protein Uptake and IgE‐Mediated Food Allergy,” Foodservice Research International 163 (2023): 112150. 10.1016/j.foodres.2022.112150 36596102

[118] M. D. Kulis , J. M. Smeekens , R. M. Immormino , and T. P. Moran , “The Airway as a Route of Sensitization to Peanut: An Update to the Dual Allergen Exposure Hypothesis,” Journal of Allergy and Clinical Immunology 148, no. 3 (2021): 689–693, https://pubmed.ncbi.nlm.nih.gov/34111450/. 34111450 10.1016/j.jaci.2021.05.035 PMC8429226

[119] W. Dijk , C. Villa , S. Benedé , et al., “Critical Features of an In Vitro Intestinal Absorption Model to Study the First Key Aspects Underlying Food Allergen Sensitization,” Comprehensive Reviews in Food Science and Food Safety 22, no. 2 (2023): 971–1005, 10.1111/1541-4337.13097. 36546415

[120] K. M. Järvinen , G. N. Konstantinou , M. Pilapil , et al., “Intestinal Permeability in Children With Food Allergy on Specific Elimination Diets,” Pediatric Allergy and Immunology 24, no. 6 (2013): 589–595, 10.1111/pai.12106. 23909601 PMC3774110

[121] C. Gomez‐Casado , J. Sanchez‐Solares , E. Izquierdo , A. Díaz‐Perales , D. Barber , and M. M. Escribese , “Oral Mucosa as a Potential Site for Diagnosis and Treatment of Allergic and Autoimmune Diseases,” Food 10, no. 5 (2021): 970, https://www.mdpi.com/2304‐8158/10/5/970/htm. 10.3390/foods10050970 PMC8146604 33925074

[122] E. Mazzinelli , I. Favuzzi , A. Arcovito , et al., “Oral Mucosa Models to Evaluate Drug Permeability,” Pharmaceutics 15, no. 5 (2023): 1559, https://www.mdpi.com/1999‐4923/15/5/1559/htm. 37242801 10.3390/pharmaceutics15051559 PMC10220859

[123] C. G. Dirks , M. H. Pedersen , M. H. Platzer , C. Bindslev‐Jensen , P. S. Skov , and L. K. Poulsen , “Does Absorption Across the Buccal Mucosa Explain Early Onset of Food‐Induced Allergic Systemic Reactions?,” Journal of Allergy and Clinical Immunology 115, no. 6 (2005): 1321–1323, https://linkinghub.elsevier.com/retrieve/pii/S0091674905006044. 15940158 10.1016/j.jaci.2005.03.027

[124] A. Pahlow Mose , E. Mortz , P. Stahl Skov , et al., “The Quest for Ingested Peanut Protein in Human Serum,” Allergy 75, no. 7 (2020): 1721–1729. 31715004 10.1111/all.14109

[125] A. P. Mose , C. G. Mortz , E. Eller , U. Sprogøe , T. Barington , and C. Bindslev‐Jensen , “Dose‐Time‐Response Relationship in Peanut Allergy Using a Human Model of Passive Cutaneous Anaphylaxis,” Journal of Allergy and Clinical Immunology 139, no. 6 (2017): 2015–2016.e4. 28189335 10.1016/j.jaci.2016.11.034

[126] H. Bernard , P. J. Turner , S. Ah‐Leung , M. Ruiz‐Garcia , E. N. Clare Mills , and K. Adel‐Patient , “Circulating Ara h 6 as a Marker of Peanut Protein Absorption in Tolerant and Allergic Humans Following Ingestion of Peanut‐Containing Foods,” Clinical & Experimental Allergy 50, no. 9 (2020): 1093–1102, 10.1111/cea.13706. 32648641

[127] B. A. Williams , Y. Guo , L. Soller , E. S. Chan , and A. Pratap‐Singh , “Total Protein Content and In Vitro Protein Penetration Rates of Sublingual Immunotherapy Preparations,” Annals of Allergy, Asthma & Immunology 133, no. 6 (2024): 723–725. 10.1016/j.anai.2024.07.033 39127386

[128] S. J. Koppelman , M. Smits , M. Tomassen , et al., “Release of Major Peanut Allergens From Their Matrix Under Various pH and Simulated Saliva Conditions‐Ara h2 and Ara h6 Are Readily Bio‐Accessible,” Nutrients 10, no. 9 (2018): 1281, https://pubmed.ncbi.nlm.nih.gov/30208580/. 30208580 10.3390/nu10091281 PMC6165493

[129] H. S. Porterfield , K. S. Murray , D. G. Schlichting , et al., “Effector Activity of Peanut Allergens: A Critical Role for Ara h 2, Ara h 6, and Their Variants,” Clinical & Experimental Allergy 39, no. 7 (2009): 1099–1108, 10.1111/j.1365-2222.2009.03273.x. 19438581 PMC2752635

[130] M. Kulis , X. Chen , J. Lew , et al., “The 2S Albumin Allergens of *Arachis hypogaea*, Ara h 2 and Ara h 6, Are the Major Elicitors of Anaphylaxis and Can Effectively Desensitize Peanut‐Allergic Mice,” Clinical & Experimental Allergy 42, no. 2 (2012): 326–336, 10.1111/j.1365-2222.2011.03934.x. 22288514 PMC3270336

[131] J. M. Smeekens , C. Baloh , N. Lim , et al., “Peanut‐Specific IgG4 and IgA in Saliva Are Modulated by Peanut Oral Immunotherapy,” Journal of Allergy and Clinical Immunology. In Practice 10, no. 12 (2022): 3270–3275. 35944894 10.1016/j.jaip.2022.07.030 PMC9742136

[132] E. Eller , P. Stahl Skov , K. Baumann , C. Hilger , M. Ollert , and C. Bindslev‐Jensen , “Delayed Reaction in Alpha‐Gal Allergy Is Reflected in Serum Levels After Ingestion of Pork Kidney, and Absorption Is Dependent on Food Processing,” Clinical and Experimental Allergy 52, no. 1 (2022): 197–200. 34779547 10.1111/cea.14054

[133] H. Maeda , S. Ichimizu , H. Watanabe , et al., “Cell‐Penetrating Albumin Enhances the Sublingual Delivery of Antigens Through Macropinocytosis,” International Journal of Biological Macromolecules 221 (2022): 1439–1452. 36126807 10.1016/j.ijbiomac.2022.09.132

[134] D. Barber , F. De La Torre , M. Lombardero , et al., “Component‐Resolved Diagnosis of Pollen Allergy Based on Skin Testing With Profilin, Polcalcin and Lipid Transfer Protein Pan‐Allergens,” Clinical & Experimental Allergy 39, no. 11 (2009): 1764–1773, 10.1111/j.1365-2222.2009.03351.x. 19877313

[135] C. Mastrorilli , S. Tripodi , C. Caffarelli , et al., “Endotypes of Pollen‐Food Syndrome in Children With Seasonal Allergic Rhinoconjunctivitis: A Molecular Classification,” Allergy 71, no. 8 (2016): 1181–1191, 10.1111/all.12888. 26999633

[136] R. Asero , S. Tripodi , A. Dondi , et al., “Prevalence and Clinical Relevance of IgE Sensitization to Profilin in Childhood: A Multicenter Study,” International Archives of Allergy and Immunology 168, no. 1 (2015): 25–31, 10.1159/000441222. 26528861

[137] A. Santos and R. Van Ree , “Profilins: Mimickers of Allergy or Relevant Allergens?,” International Archives of Allergy and Immunology 155, no. 3 (2011): 191–204, 10.1159/000321178. 21293140

[138] C. Porsbjerg , K. Baines , P. Gibson , et al., “IL‐33 Is Related to Innate Immune Activation and Sensitization to HDM in Mild Steroid‐Free Asthma,” Clinical & Experimental Allergy 46, no. 4 (2016): 564–574, 10.1111/cea.12702. 26748611

[139] G. D. Rak , L. C. Osborne , M. C. Siracusa , et al., “IL‐33‐Dependent Group 2 Innate Lymphoid Cells Promote Cutaneous Wound Healing,” Journal of Investigative Dermatology 136, no. 2 (2016): 487–496. 26802241 10.1038/JID.2015.406 PMC4731037

[140] I. Martinez‐Gonzalez , C. A. Steer , and F. Takei , “Lung ILC2s Link Innate and Adaptive Responses in Allergic Inflammation,” Trends in Immunology 36, no. 3 (2015): 189–195. 25704560 10.1016/j.it.2015.01.005

[141] D. Rosace , C. Gomez‐Casado , P. Fernandez , et al., “Profilin‐Mediated Food‐Induced Allergic Reactions Are Associated With Oral Epithelial Remodeling,” Journal of Allergy and Clinical Immunology 143, no. 2 (2019): 681–690.e1, https://pubmed.ncbi.nlm.nih.gov/29705246/. 29705246 10.1016/j.jaci.2018.03.013

[142] R. Doucet‐Ladevèze , S. Holvoet , F. Raymond , et al., “Transcriptomic Analysis Links Eosinophilic Esophagitis and Atopic Dermatitis,” Frontiers in Pediatrics 7 (2019): 492012, www.frontiersin.org. 10.3389/fped.2019.00467 PMC6879454 31824894

[143] J. Tulyeu , H. Kumagai , E. Jimbo , et al., “Probiotics Prevents Sensitization to Oral Antigen and Subsequent Increases in Intestinal Tight Junction Permeability in Juvenile–Young Adult Rats,” Microorganisms 7, no. 10 (2019): 463, https://www.mdpi.com/2076‐2607/7/10/463/htm. 31623229 10.3390/microorganisms7100463 PMC6843414

[144] B. Filipiak‐Pittroff , C. Schnopp , D. Berdel , et al., “Predictive Value of Food Sensitization and Filaggrin Mutations in Children With Eczema,” Journal of Allergy and Clinical Immunology 128, no. 6 (2011): 1235–1241.e5, https://pubmed.ncbi.nlm.nih.gov/22030464/. 22030464 10.1016/j.jaci.2011.09.014

[145] V. A. Ferraro , S. Zanconato , and S. Carraro , “The Epithelial Barrier Hypothesis in Food Allergies: The State of the Art,” Nutrients 17, no. 6 (2025): 1014, https://www.mdpi.com/2072‐6643/17/6/1014/htm. 40290033 10.3390/nu17061014 PMC11944793

[146] R. Paganelli and R. J. Levinsky , “Differences in Specific Antibody Responses of Human Tonsillar Cells to an Oral and a Parenteral Antigen,” Scandinavian Journal of Immunology 14, no. 4 (1981): 353–358, 10.1111/j.1365-3083.1981.tb00575.x. 7336168

[147] J. B. de Albuquerque , L. M. Altenburger , J. Abe , et al., “Microbial Uptake in Oral Mucosa‐Draining Lymph Nodes Leads to Rapid Release of Cytotoxic CD8+ T Cells Lacking a Gut‐Homing Phenotype,” Science Immunology 7, no. 72 (2022): eabf1861, 10.1126/sciimmunol.abf1861. 35714202

[148] T. Gu , W. Zhang , L. Tan , et al., “Intratonsillar Immunotherapy: A Convenient and Effective Alternative to Subcutaneous Immunotherapy for Allergic Rhinitis,” Research 8 (2025): 0573, https://pmc.ncbi.nlm.nih.gov/articles/PMC11735709/. 39822282 10.34133/research.0573 PMC11735709

[149] S. G. Byars , S. C. Stearns , and J. J. Boomsma , “Association of Long‐Term Risk of Respiratory, Allergic, and Infectious Diseases With Removal of Adenoids and Tonsils in Childhood,” JAMA Otolaryngology. Head & Neck Surgery 144, no. 7 (2018): 594–603, https://jamanetwork.com/journals/jamaotolaryngology/fullarticle/2683621. 29879264 10.1001/jamaoto.2018.0614 PMC6145787

[150] T. Hanif , L. E. Ivaska , F. Ahmad , et al., “Tonsillar Transcriptional Profiles in Atopic and Non‐Atopic Subjects,” Allergy 78, no. 2 (2023): 522–536. 35899482 10.1111/all.15458 PMC10087516

[151] J. Yuan , P. Tong , X. Meng , et al., “Oral Exposure to *Staphylococcus aureus* Enterotoxin B Could Promote the Ovalbumin‐Induced Food Allergy by Enhancing the Activation of DCs and T Cells,” Frontiers in Immunology 14 (2023): 1250458. 37908363 10.3389/fimmu.2023.1250458 PMC10615071

[152] P. Korosec , P. J. Turner , M. Silar , et al., “Basophils, High‐Affinity IgE Receptors, and CCL2 in Human Anaphylaxis,” Journal of Allergy and Clinical Immunology 140, no. 3 (2017): 750–758.e15, https://linkinghub.elsevier.com/retrieve/pii/S0091674917304232. 28342911 10.1016/j.jaci.2016.12.989 PMC5587023

[153] K. Nishida , S. Yamasaki , Y. Ito , et al., “FcεRI‐Mediated Mast Cell Degranulation Requires Calcium‐Independent Microtubule‐Dependent Translocation of Granules to the Plasma Membrane,” Journal of Cell Biology 170, no. 1 (2005): 115–126. 15998803 10.1083/jcb.200501111 PMC2171390

[154] D. MacGlashan , “IgE and FceRI Regulation,” Clinical Reviews in Allergy and Immunology 29, no. 1 (2005): 49–60. 16222083 10.1385/criai:29:1:049

[155] H. J. Bax , A. H. Keeble , and H. J. Gould , “Cytokinergic IgE Action in Mast Cell Activation,” Frontiers in Immunology 3 (2012): 229. 22888332 10.3389/fimmu.2012.00229 PMC3412263

[156] B. M. Vonakis , H. Chen , H. Haleem‐Smith , and H. Metzger , “The Unique Domain as the Site on Lyn Kinase for Its Constitutive Association With the High Affinity Receptor for IgE,” Journal of Biological Chemistry 272, no. 38 (1997): 24072–24080. 9295361 10.1074/jbc.272.38.24072

[157] V. S. Pribluda , C. Pribluda , and H. Metzger , “Transphosphorylation as the Mechanism by Which the High‐Affinity Receptor for IgE Is Phosphorylated Upon Aggregation,” National Academy of Sciences of the United States of America 91, no. 23 (1994): 11246–11250. 10.1073/pnas.91.23.11246 PMC45204 7526393

[158] S. Lin , C. Cicala , A. M. Scharenberg , and J. P. Kinet , “The FcεRIβ Subunit Functions as an Amplifier of FcεRIγ‐Mediated Cell Activation Signals,” Cell 85, no. 7 (1996): 985–995. 8674126 10.1016/s0092-8674(00)81300-8

[159] P. S. Costello , M. Turner , A. E. Walters , et al., “Critical Role for the Tyrosine Kinase Syk in Signalling Through the High Affinity IgE Receptor of Mast Cells,” Oncogene 13, no. 12 (1996): 2595–2605. 9000133

[160] A. M. Scharenberg , S. Lin , B. Cuenod , H. Yamamura , and J. P. Kinet , “Reconstitution of Interactions Between Tyrosine Kinases and the High Affinity IgE Receptor Which Are Controlled by Receptor Clustering,” EMBO Journal 14, no. 14 (1995): 3385–3394. 7628439 10.1002/j.1460-2075.1995.tb07344.x PMC394405

[161] D. J. Rawlings , A. M. Scharenberg , H. Park , et al., “Activation of BTK by a Phosphorylation Mechanism Initiated by SRC Family Kinases,” Science (1979) 271, no. 5250 (1996): 822–825. 10.1126/science.271.5250.822 8629002

[162] M. Hoth and R. Penner , “Depletion of Intracellular Calcium Stores Activates a Calcium Current in Mast Cells,” Nature 355, no. 6358 (1992): 353–356. 1309940 10.1038/355353a0

[163] M. Gulliksson , L. Palmberg , G. Nilsson , S. Ahlstedt , and M. Kumlin , “Release of Prostaglandin D_2_ and Leukotriene C_4_ in Response to Hyperosmolar Stimulation of Mast Cells,” Allergy 61, no. 12 (2006): 1473–1479. 17073880 10.1111/j.1398-9995.2006.01213.x

[164] D. MacGlashan and J. Warner , “Stimulus‐Dependent Leukotriene Release From Human Basophils: A Comparative Study of C5a and Fmet‐Leu‐Phe,” Journal of Leukocyte Biology 49, no. 1 (1991): 29–40, https://academic.oup.com/jleukbio/article/49/1/29/6977549. 1898612 10.1002/jlb.49.1.29

[165] B. Ochensberger , S. Rihs , T. Brunner , and C. A. Dahinden , “IgE‐Independent Interleukin‐4 Expression and Induction of a Late Phase of Leukotriene C4 Formation in Human Blood Basophils,” Blood 86, no. 11 (1995): 4039–4049. 7492759

[166] C. M. Hogaboam , D. Donigi‐Gale , T. S. Shoupe , E. Y. Bissonnette , A. D. Befus , and J. L. Wallace , “Platelet‐Activating Factor Synthesis by Peritoneal Mast Cells and Its Inhibition by Two Quinoline‐Based Compounds,” British Journal of Pharmacology 105, no. 1 (1992): 87–92. 1596692 10.1111/j.1476-5381.1992.tb14215.x PMC1908618

[167] N. Nakatani , N. Uozumi , K. Kume , M. Murakami , I. Kudo , and T. Shimizu , “Role of Cytosolic Phospholipase A2 in the Production of Lipid Mediators and Histamine Release in Mouse Bone‐Marrow‐Derived Mast Cells,” Biochemical Journal 35, no. Pt 2 (2000): 311–317. PMC1221461 11085923

[168] D. Kempuraj , B. Madhappan , S. Christodoulou , et al., “Flavonols Inhibit Proinflammatory Mediator Release, Intracellular Calcium Ion Levels and Protein Kinase C Theta Phosphorylation in Human Mast Cells,” British Journal of Pharmacology 145, no. 7 (2005): 934–944. 15912140 10.1038/sj.bjp.0706246 PMC1576204

[169] K. Kandere‐Grzybowska , D. Kempuraj , J. Cao , C. L. Cetrulo , and T. C. Theoharides , “Regulation of IL‐1‐Induced Selective IL‐6 Release From Human Mast Cells and Inhibition by Quercetin,” British Journal of Pharmacology 148, no. 2 (2006): 208–215. 16532021 10.1038/sj.bjp.0706695 PMC1617055

[170] T. Pecaric‐Petkovic , S. A. Didichenko , S. Kaempfer , N. Spiegl , and C. A. Dahinden , “Human Basophils and Eosinophils Are the Direct Target Leukocytes of the Novel IL‐1 Family Member IL‐33,” Blood 113, no. 7 (2009): 1526–1534. 18955562 10.1182/blood-2008-05-157818 PMC2644080

[171] M. A. Bawazeer and T. C. Theoharides , “IL‐33 Stimulates Human Mast Cell Release of CCL5 and CCL2 via MAPK and NF‐κB, Inhibited by Methoxyluteolin,” European Journal of Pharmacology 865 (2019): 172760. 31669588 10.1016/j.ejphar.2019.172760

[172] S. Roy , C. Chompunud Na Ayudhya , M. Thapaliya , V. Deepak , and H. Ali , “Multifaceted MRGPRX2: New Insight Into the Role of Mast Cells in Health and Disease,” Journal of Allergy and Clinical Immunology 148, no. 2 (2021): 293–308, https://linkinghub.elsevier.com/retrieve/pii/S0091674921007211. 33957166 10.1016/j.jaci.2021.03.049 PMC8355064

[173] H. C. Oettgen , “Mast Cells in Food Allergy: Inducing Immediate Reactions and Shaping Long‐Term Immunity,” Journal of Allergy and Clinical Immunology 151, no. 1 (2023): 21–25. 36328809 10.1016/j.jaci.2022.10.003

[174] E. F. Knol , T. W. Kuijpers , F. P. Mul , and D. Roos , “Stimulation of Human Basophils Results in Homotypic Aggregation. A Response Independent of Degranulation,” Journal of Immunology 151, no. 9 (1993): 4926–4933, http://www.ncbi.nlm.nih.gov/pubmed/7691958. 7691958

[175] B. Bochner , D. W. J. MacGlashan , G. Marcotte , and R. P. Schleimer , “IgE‐Dependent Regulation of Human Basophil Adherence to Vascular Endothelium,” Journal of Immunology 142, no. 9 (1989): 3180–3186. 2468714

[176] B. S. Bochner , S. A. Sterbinsky , E. F. Knol , et al., “Function and Expression of Adhesion Molecules on Human Basophils,” Journal of Allergy and Clinical Immunology 94, no. 6 (1994): 1157–1162, https://linkinghub.elsevier.com/retrieve/pii/0091674994903263. 7798554 10.1016/0091-6749(94)90326-3

[177] L. Tordesillas , A. H. Rahman , B. M. Hartmann , H. A. Sampson , and M. C. Berin , “Mass Cytometry Profiling the Response of Basophils and the Complete Peripheral Blood Compartment to Peanut,” Journal of Allergy and Clinical Immunology 138, no. 6 (2016): 1741–1744.e9. 27531074 10.1016/j.jaci.2016.06.048 PMC5148641

[178] C. T. Watson , A. T. Cohain , R. S. Griffin , et al., “Integrative Transcriptomic Analysis Reveals Key Drivers of Acute Peanut Allergic Reactions,” Nature Communications 8, no. 1 (2017): 1943. 10.1038/s41467-017-02188-7 PMC5715016 29203772

[179] A. N. Do , C. T. Watson , A. T. Cohain , et al., “Dual Transcriptomic and Epigenomic Study of Reaction Severity in Peanut‐Allergic Children,” Journal of Allergy and Clinical Immunology 145, no. 4 (2020): 1219–1230. 31838046 10.1016/j.jaci.2019.10.040 PMC7192362

[180] L. Argiz , M. Valsami‐Fokianos , S. Arasi , et al., “Clinical‐Hematological Changes and Predictors of Severity in Acute Food Protein–Induced Enterocolitis Syndrome Reactions at Oral Food Challenge: A Multicenter Observational Study,” Journal of Allergy and Clinical Immunology: In Practice 12, no. 9 (2024): 2454–2467.e8. 38796100 10.1016/j.jaip.2024.05.024

[181] S. B. Sindher , K. C. Nadeau , R. S. Chinthrajah , et al., “Efficacy and Safety of Dupilumab in Children With Peanut Allergy: A Multicenter, Open‐Label, Phase II Study,” Allergy 80, no. 1 (2025): 227–237. 39673452 10.1111/all.16404 PMC11724241

[182] R. S. Chinthrajah , S. B. Sindher , K. C. Nadeau , et al., “Dupilumab as an Adjunct to Oral Immunotherapy in Pediatric Patients With Peanut Allergy,” Allergy 80, no. 3 (2025): 827–842. 39673367 10.1111/all.16420 PMC11891407

[183] J. Klueber , R. Czolk , F. Codreanu‐Morel , et al., “High‐Dimensional Immune Profiles Correlate With Phenotypes of Peanut Allergy During Food‐Allergic Reactions,” Allergy 78, no. 4 (2023): 1020–1035. 35700055 10.1111/all.15408

[184] I. Suhrkamp , A. Scheffold , and G. Heine , “T‐Cell Subsets in Allergy and Tolerance Induction,” European Journal of Immunology 53, no. 10 (2023): 2249983, 10.1002/eji.202249983. 37489248

[185] D. Lozano‐Ojalvo , S. R. Tyler , C. J. Aranda , et al., “Allergen Recognition by Specific Effector Th2 Cells Enables IL ‐2‐Dependent Activation of Regulatory T‐Cell Responses in Humans,” Allergy 78, no. 3 (2023): 697–713. 36089900 10.1111/all.15512 PMC10111618

[186] A. Renand , M. Farrington , E. Whalen , et al., “Heterogeneity of Ara h Component‐Specific CD4 T Cell Responses in Peanut‐Allergic Subjects,” Frontiers in Immunology 9 (2018): 1408. 29988522 10.3389/fimmu.2018.01408 PMC6026622

[187] K. A. Weissler , M. Rasooly , T. DiMaggio , et al., “Identification and Analysis of Peanut‐Specific Effector T and Regulatory T Cells in Children Allergic and Tolerant to Peanut,” Journal of Allergy and Clinical Immunology 141, no. 5 (2018): 1699–1710.e7. 29454004 10.1016/j.jaci.2018.01.035 PMC5938104

[188] B. Ruiter , N. P. Smith , B. Monian , et al., “Expansion of the CD4+ Effector T‐Cell Repertoire Characterizes Peanut‐Allergic Patients With Heightened Clinical Sensitivity,” Journal of Allergy and Clinical Immunology 145, no. 1 (2020): 270–282. 31654649 10.1016/j.jaci.2019.09.033 PMC6949413

[189] M. R. Neeland , S. Andorf , M. Manohar , et al., “Mass Cytometry Reveals Cellular Fingerprint Associated With IgE+ Peanut Tolerance and Allergy in Early Life,” Nature Communications 11, no. 1 (2020): 1091. 10.1038/s41467-020-14919-4 PMC7046671 32107388

[190] N. Qamar , A. B. Fishbein , K. A. Erickson , et al., “Naturally Occurring Tolerance Acquisition to Foods in Previously Allergic Children Is Characterized by Antigen Specificity and Associated With Increased Subsets of Regulatory T Cells,” Clinical and Experimental Allergy 45, no. 11 (2015): 1663–1672. 25989379 10.1111/cea.12570 PMC4607624

[191] A. Syed , M. A. Garcia , S. C. Lyu , et al., “Peanut Oral Immunotherapy Results in Increased Antigen‐Induced Regulatory T‐Cell Function and Hypomethylation of Forkhead Box Protein 3 (FOXP3),” Journal of Allergy and Clinical Immunology 133, no. 2 (2014): 500–510.e11, https://linkinghub.elsevier.com/retrieve/pii/S0091674913029576. 24636474 10.1016/j.jaci.2013.12.1037 PMC4121175

[192] V. Turcanu , S. J. Maleki , and G. Lack , “Characterization of Lymphocyte Responses to Peanuts in Normal Children, Peanut‐Allergic Children, and Allergic Children Who Acquired Tolerance to Peanuts,” Journal of Clinical Investigation 111, no. 7 (2003): 1065–1072, http://www.jci.org/articles/view/16142. 12671056 10.1172/JCI16142 PMC152580

[193] T. Eiwegger , N. Rigby , L. Mondoulet , et al., “Gastro‐Duodenal Digestion Products of the Major Peanut Allergen Ara h 1 Retain an Allergenic Potential,” Clinical & Experimental Allergy 36, no. 10 (2006): 1281–1288, 10.1111/j.1365-2222.2006.02565.x. 17014437

[194] M. Bublin , M. Kostadinova , C. Radauer , et al., “Engineering of Structural Variants of the Major Peanut Allergens Ara h 2 and Ara h 6 for Allergen‐Specific Immunotherapy,” Journal of Allergy and Clinical Immunology 143, no. 3 (2019): 1226–1229.e10, https://linkinghub.elsevier.com/retrieve/pii/S0091674918315811. 30414861 10.1016/j.jaci.2018.10.039

[195] J. Calise , H. DeBerg , N. Garabatos , et al., “Distinct Trajectories Distinguish Antigen‐Specific T Cells in Peanut‐Allergic Individuals Undergoing Oral Immunotherapy,” Journal of Allergy and Clinical Immunology 152, no. 1 (2023): 155–166.e9. 37003475 10.1016/j.jaci.2023.03.020 PMC10330178

[196] S. S. Tay , A. T. Clark , J. Deighton , Y. King , and P. W. Ewan , “T Cell Proliferation and Cytokine Responses to Ovalbumin and Ovomucoid Detected in Children With and Without Egg Allergy,” Clinical and Experimental Allergy 37, no. 10 (2007): 1519–1527. 17883731 10.1111/j.1365-2222.2007.02807.x

[197] L. De Vlieger , L. Nuyttens , C. Keppens , et al., “Egg Allergen‐Specific T‐Cell and Cytokine Responses in Healthy and Egg‐Allergic Children Naturally Tolerating Baked Egg,” Pediatric Allergy and Immunology 36, no. 1 (2025): e70018, 10.1111/pai.70018. 39803990

[198] J. Lee , T. Yamamoto , S. Hayashi , H. Kuramoto , and M. Kadowaki , “Enhancement of CGRP Sensory Afferent Innervation in the Gut During the Development of Food Allergy in an Experimental Murine Model,” Biochemical and Biophysical Research Communications 430, no. 3 (2013): 895–900, https://pubmed.ncbi.nlm.nih.gov/23261435/. 23261435 10.1016/j.bbrc.2012.12.058

[199] T. Yashiro , H. Ogata , S. F. Zaidi , et al., “Pathophysiological Roles of Neuro‐Immune Interactions Between Enteric Neurons and Mucosal Mast Cells in the Gut of Food Allergy Mice,” Cells 10, no. 7 (2021): 1586, https://pubmed.ncbi.nlm.nih.gov/34201851/. 34201851 10.3390/cells10071586 PMC8305700

[200] A. D. Fryer , L. H. Stein , Z. Nie , et al., “Neuronal Eotaxin and the Effects of CCR3 Antagonist on Airway Hyperreactivity and M2 Receptor Dysfunction,” Journal of Clinical Investigation 116, no. 1 (2006): 228–236. 16374515 10.1172/JCI25423 PMC1319219

[201] M. A. Thornton , N. Akasheh , M. T. Walsh , et al., “Eosinophil Recruitment to Nasal Nerves After Allergen Challenge in Allergic Rhinitis,” Clinical Immunology 147, no. 1 (2013): 50–57, https://pubmed.ncbi.nlm.nih.gov/23518598/. 23518598 10.1016/j.clim.2013.02.008

[202] W. Jiang , M. E. Kreis , C. Eastwood , A. J. Kirkup , P. P. A. Humphrey , and D. Grundy , “5‐HT3 and Histamine H± Receptors Mediate Afferent Nerve Sensitivity to Intestinal Anaphylaxis in Rats,” Gastroenterology 119, no. 5 (2000): 1267–1275, https://pubmed.ncbi.nlm.nih.gov/11054384/. 11054384 10.1053/gast.2000.19461

[203] M. Steinhoff , N. Vergnolle , S. H. Young , et al., “Agonists of Proteinase‐Activated Receptor 2 Induce Inflammation by a Neurogenic Mechanism,” Nature Medicine 6, no. 2 (2000): 151–158, https://pubmed.ncbi.nlm.nih.gov/10655102/. 10.1038/72247 10655102

[204] C. Bao , O. Chen , H. Sheng , et al., “A Mast Cell‐Thermoregulatory Neuron Circuit Axis Regulates Hypothermia in Anaphylaxis,” Science Immunology 8, no. 81 (2023): eadc9417, https://pubmed.ncbi.nlm.nih.gov/36930731/. 36930731 10.1126/sciimmunol.adc9417 PMC10331449

[205] T. Plum , R. Binzberger , R. Thiele , et al., “Mast Cells Link Immune Sensing to Antigen‐Avoidance Behaviour,” Nature 620, no. 7974 (2023): 634–642, https://www.nature.com/articles/s41586‐023‐06188‐0. 37438525 10.1038/s41586-023-06188-0 PMC10432277

[206] E. B. Florsheim , N. D. Bachtel , J. L. Cullen , et al., “Immune Sensing of Food Allergens Promotes Avoidance Behaviour,” Nature 620, no. 7974 (2023): 643–650, https://www.nature.com/articles/s41586‐023‐06362‐4. 37437602 10.1038/s41586-023-06362-4 PMC10432274

[207] J. M. Cyphert , M. Kovarova , I. C. Allen , et al., “Cooperation Between Mast Cells and Neurons Is Essential for Antigen‐Mediated Bronchoconstriction,” Journal of Immunology (Baltimore, Md.: 1950) 182, no. 12 (2009): 7430–7439, https://pubmed.ncbi.nlm.nih.gov/19494266/. 19494266 10.4049/jimmunol.0900039 PMC3901060

[208] G. Bosmans , I. Appeltans , N. Stakenborg , et al., “Vagus Nerve Stimulation Dampens Intestinal Inflammation in a Murine Model of Experimental Food Allergy,” Allergy 74, no. 9 (2019): 1748–1759, https://pubmed.ncbi.nlm.nih.gov/30897213/. 30897213 10.1111/all.13790 PMC6790670

[209] T. Yamamoto , T. Kodama , J. Lee , et al., “Anti‐Allergic Role of Cholinergic Neuronal Pathway via α7 Nicotinic ACh Receptors on Mucosal Mast Cells in a Murine Food Allergy Model,” PLoS One 9, no. 1 (2014): e85888, https://pubmed.ncbi.nlm.nih.gov/24454942/. 24454942 10.1371/journal.pone.0085888 PMC3894205

[210] H. van der Kleij , N. Charles , K. Karimi , et al., “Evidence for Neuronal Expression of Functional Fc (ε{Lunate} and γ) Receptors,” Journal of Allergy and Clinical Immunology 125, no. 3 (2010): 757–760, https://pubmed.ncbi.nlm.nih.gov/20132972/. 20132972 10.1016/j.jaci.2009.10.054 PMC2839037

[211] C. Perner , C. H. Flayer , X. Zhu , et al., “Substance P Release by Sensory Neurons Triggers Dendritic Cell Migration and Initiates the Type‐2 Immune Response to Allergens,” Immunity 53, no. 5 (2020): 1063–1077.e7, https://pubmed.ncbi.nlm.nih.gov/33098765/. 33098765 10.1016/j.immuni.2020.10.001 PMC7677179

[212] C. H. Flayer , I. J. Kernin , P. R. Matatia , et al., “A γδ T Cell‐IL‐3 Axis Controls Allergic Responses Through Sensory Neurons,” Nature 634, no. 8033 (2024): 440–446, https://pubmed.ncbi.nlm.nih.gov/39232162/. 39232162 10.1038/s41586-024-07869-0 PMC12051158

[213] L. Duan , A. Celik , J. A. Hoang , et al., “Basophil Activation Test Shows High Accuracy in the Diagnosis of Peanut and Tree Nut Allergy: The Markers of Nut Allergy Study,” Allergy 76, no. 6 (2021): 1800–1812. 33300157 10.1111/all.14695 PMC8608143

[214] W. G. Shreffler , “Evaluation of Basophil Activation in Food Allergy: Present and Future Applications,” Current Opinion in Allergy and Clinical Immunology 6, no. 3 (2006): 226–233, https://pubmed.ncbi.nlm.nih.gov/16670519/. 16670519 10.1097/01.all.0000225165.83144.2f

[215] A. F. Santos , O. Alpan , and H. J. Hoffmann , “Basophil Activation Test: Mechanisms and Considerations for Use in Clinical Trials and Clinical Practice,” Allergy 76, no. 8 (2021): 2420–2432, 10.1111/all.14747. 33475181

[216] D. Giulia , D. F. Paola , D. L. Armando , et al., “Applications of Basophil Activation Test in Paediatric Allergic Diseases,” World Allergy Organization Journal 17, no. 12 (2024): 100998. 39734398 10.1016/j.waojou.2024.100998 PMC11681913

[217] J. Folkerts , N. Gaudenzio , M. Maurer , et al., “Rapid Identification of Human Mast Cell Degranulation Regulators Using Functional Genomics Coupled to High‐Resolution Confocal Microscopy,” Nature Protocols 15 (2020): 1285–1310, 10.1038/s41596-019-0288-6. 32060492 PMC7197894

[218] M. Farazuddin , N. Ludka , and J. Baker , “The Use of LAD2 Cells in the Mast Cell Activation Test (MAT): A Potential Tool for Food Allergy Diagnosis,” Journal of Allergy and Clinical Immunology 147, no. 2 (2021): AB88, https://www.jacionline.org/action/showFullText?pii=S0091674920320996.

[219] A. S. Kirshenbaum , Y. Yin , J. Bruce Sundstrom , G. Bandara , and D. D. Metcalfe , “Description and Characterization of a Novel Human Mast Cell Line for Scientific Study,” International Journal of Molecular Sciences 20, no. 22 (2019): 5520, https://pubmed.ncbi.nlm.nih.gov/31698677/. 31698677 10.3390/ijms20225520 PMC6888318

[220] N. Zbären , D. Brigger , D. Bachmann , et al., “A Novel Functional Mast Cell Assay for the Detection of Allergies,” Journal of Allergy and Clinical Immunology 149, no. 3 (2022): 1018–1030.e11, https://www.jacionline.org/action/showFullText?pii=S009167492101246X. 34418424 10.1016/j.jaci.2021.08.006

[221] A. F. Santos , N. Couto‐Francisco , N. Bécares , M. Kwok , H. T. Bahnson , and G. Lack , “A Novel Human Mast Cell Activation Test for Peanut Allergy,” Journal of Allergy and Clinical Immunology 142, no. 2 (2018): 689–691.e9, https://www.jacionline.org/action/showFullText?pii=S0091674918305189. 29731128 10.1016/j.jaci.2018.03.011 PMC6080741

[222] N. Bachmeier‐Zbären , A. Celik , R. van Brummelen , et al., “Clinical Utility Analysis of the Hoxb8 Mast Cell Activation Test for the Diagnosis of Peanut Allergy,” Allergy: European Journal of Allergy and Clinical Immunology 1 (2024): 215–226. 10.1111/all.16341 PMC11724244 39340441

[223] P. Stoffersen , P. S. Skov , L. K. Poulsen , and B. M. Jensen , “The Allergen‐Specific IgE Concentration Is Important for Optimal Histamine Release From Passively Sensitized Basophils,” Frontiers in Allergy 3 (2022): 875119, www.frontiersin.org. 35769579 10.3389/falgy.2022.875119 PMC9234936

[224] D. G. Ebo , C. H. Bridts , C. H. Mertens , and V. Sabato , “Current Perspectives Principles, Potential, and Limitations of Ex Vivo Basophil Activation by Flow Cytometry in Allergology: A Narrative Review,” Journal of Allergy and Clinical Immunology 147 (2021): 1143–1153, 10.1016/j.jaci.2020.10.027. 33152367

[225] L. F. Larsen , N. Juel‐Berg , K. S. Hansen , et al., “A Comparative Study on Basophil Activation Test, Histamine Release Assay, and Passive Sensitization Histamine Release Assay in the Diagnosis of Peanut Allergy,” Allergy: European Journal of Allergy and Clinical Immunology 73, no. 1 (2018): 137–144. 28686296 10.1111/all.13243

[226] D. G. Ebo , R. Bahri , A. Eggel , V. Sabato , C. Tontini , and J. Elst , “Flow Cytometry‐Based Basophil and Mast Cell Activation Tests in Allergology: State of the Art,” Journal of Allergy and Clinical Immunology 155, no. 2 (2025): 286–297, https://pubmed.ncbi.nlm.nih.gov/39581294/. 39581294 10.1016/j.jaci.2024.11.023

[227] J. Santos , C. Bayarri , E. Saperas , et al., “Characterisation of Immune Mediator Release During the Immediate Response to Segmental Mucosal Challenge in the Jejunum of Patients With Food Allergy,” Gut 45, no. 4 (1999): 553–558, 10.1136/gut.45.4.553. 10486364 PMC1727679

[228] S. C. Bischoff , J. Mayer , P. N. Meier , G. Zeck‐Kapp , and M. P. Manns , “Clinical Significance of the Colonoscopic Allergen Provocation Test,” International Archives of Allergy and Immunology 113, no. 1–3 (1997): 348–351, 10.1159/000237598. 9130574

[229] L. Hung , A. Celik , X. Yin , et al., “Precision Cut Intestinal Slices, a Novel Model of Acute Food Allergic Reactions,” Allergy 15, no. 2 (2022): 17. 10.1111/all.15579 PMC10098956 36377289

[230] A. Wohlsen , C. Martin , E. Vollmer , et al., “The Early Allergic Response in Small Airways of Human Precision‐Cut Lung Slices,” European Respiratory Journal 21, no. 6 (2003): 1024–1032, https://pubmed.ncbi.nlm.nih.gov/12797499/. 12797499 10.1183/09031936.03.00027502

[231] C. Koziol‐White , E. Gebski , G. Cao , and R. A. Panettieri , “Precision Cut Lung Slices: An Integrated Ex Vivo Model for Studying Lung Physiology, Pharmacology, Disease Pathogenesis and Drug Discovery,” Respiratory Research 25, no. 1 (2024): 1–21, 10.1186/s12931-024-02855-6. 38824592 PMC11144351

[232] M. Konigshoff , M. Lehmann , M. Rojas , et al., “Precision‐Cut Lung Slices: Emerging Tools for Preclinical and Translational Lung Research: An Official American Thoracic Society Workshop Report,” American Journal of Respiratory Cell and Molecular Biology 72, no. 1 (2024): 16–31, 10.1165/rcmb.2024-0479ST. PMC11707673 39499861

[233] L. Hung , H. Obernolte , K. Sewald , and T. Eiwegger , “Human Ex Vivo and In Vitro Disease Models to Study Food Allergy,” Asia Pacific Allergy 9, no. 1 (2019): e4, https://pmc.ncbi.nlm.nih.gov/articles/PMC6365658/. 30740352 10.5415/apallergy.2019.9.e4 PMC6365658

[234] V. Neuhaus , O. Danov , S. Konzok , et al., “Assessment of the Cytotoxic and Immunomodulatory Effects of Substances in Human Precision‐Cut Lung Slices,” Journal of Visualized Experiments 135 (2018): 57042, https://www.jove.com/video/57042. 10.3791/57042 PMC6101160 29806827

[235] L. Castan , K. L. Bøgh , N. Z. Maryniak , et al., “General Rights Overview of In Vivo and Ex Vivo Endpoints in Murine Food Allergy Models: Suitable for Evaluation of the Sensitizing Capacity of Novel Proteins?,” Allergy 75 (2020): 289–301, 10.1111/all.13943. 31187876 PMC7065134

[236] M. Tamari , K. Orimo , K. Motomura , et al., “The Optimal Age for Epicutaneous Sensitization Following Tape‐Stripping in BALB/c Mice,” Allergology International 67, no. 3 (2018): 380–387, https://pubmed.ncbi.nlm.nih.gov/29439856/. 29439856 10.1016/j.alit.2018.01.003

[237] M. Paolucci , V. Homère , Y. Waeckerle‐Men , et al., “Strain Matters in Mouse Models of Peanut‐Allergic Anaphylaxis: Systemic IgE‐Dependent and Ara h 2‐Dominant Sensitization in C3H Mice,” Clinical and Experimental Allergy 53, no. 5 (2023): 550–560, https://pubmed.ncbi.nlm.nih.gov/36629248/. 36629248 10.1111/cea.14279

[238] X. M. Li , B. H. Schofield , C. K. Huang , G. I. Kleiner , and H. A. Sampson , “A Murine Model of IgE‐Mediated Cow's Milk Hypersensitivity,” Journal of Allergy and Clinical Immunology 103, no. 2 II (1999): 206–214, https://www.jacionline.org/action/showFullText?pii=S0091674999704926. 9949309 10.1016/s0091-6749(99)70492-6

[239] M. C. Berin and L. Mayer , “Immunophysiology of Experimental Food Allergy,” Mucosal Immunology 2, no. 1 (2009): 24–32, https://www.nature.com/articles/mi200872. 19079331 10.1038/mi.2008.72

[240] S. Schülke and M. Albrecht , “Mouse Models for Food Allergies: Where Do we Stand?,” Cells 8, no. 6 (2019): 546, https://pmc.ncbi.nlm.nih.gov/articles/PMC6627293/. 31174293 10.3390/cells8060546 PMC6627293

[241] O. T. Burton , A. J. Stranks , J. M. Tamayo , K. J. Koleoglou , L. B. Schwartz , and H. C. Oettgen , “A Humanized Mouse Model of Anaphylactic Peanut Allergy,” Journal of Allergy and Clinical Immunology 139, no. 1 (2017): 314–322.e9, https://pubmed.ncbi.nlm.nih.gov/27417025/. 27417025 10.1016/j.jaci.2016.04.034 PMC5145786

[242] T. Feehley , C. H. Plunkett , R. Bao , et al., “Healthy Infants Harbor Intestinal Bacteria That Protect Against Food Allergy,” Nature Medicine 25, no. 3 (2019): 448–453, https://pubmed.ncbi.nlm.nih.gov/30643289/. 10.1038/s41591-018-0324-z PMC6408964 30643289

[243] A. Mauras , H. Wopereis , I. Yeop , et al., “Gut Microbiota From Infant With Cow's Milk Allergy Promotes Clinical and Immune Features of Atopy in a Murine Model,” Allergy: European Journal of Allergy and Clinical Immunology 74, no. 9 (2019): 1790–1793, https://pubmed.ncbi.nlm.nih.gov/30887528/. 30887528 10.1111/all.13787 PMC6790679

[244] J. Folkerts , F. Redegeld , G. Folkerts , et al., “Butyrate Inhibits Human Mast Cell Activation via Epigenetic Regulation of FcεRI‐Mediated Signaling,” Allergy: European Journal of Allergy and Clinical Immunology 75, no. 8 (2020): 1962–1974, https://pubmed.ncbi.nlm.nih.gov/32112426/. 10.1111/all.14254 PMC7703657 32112426

[245] B. Forde , L. Yao , R. Shaha , S. Murphy , N. Lunjani , and L. O'Mahony , “Immunomodulation by Foods and Microbes: Unravelling the Molecular Tango,” Allergy: European Journal of Allergy and Clinical Immunology 77, no. 12 (2022): 3513–3526, https://pubmed.ncbi.nlm.nih.gov/35892227/. 35892227 10.1111/all.15455 PMC10087875

[246] Y. Belkaid and T. W. Hand , “Role of the Microbiota in Immunity and Inflammation,” Cell 157, no. 1 (2014): 121, https://pmc.ncbi.nlm.nih.gov/articles/PMC4056765/. 24679531 10.1016/j.cell.2014.03.011 PMC4056765

